# IL-15 and IL-21 synergy improves anti-tumor efficacy of iPSC-derived cytotoxic T cells in solid tumors

**DOI:** 10.1016/j.ymthe.2026.04.036

**Published:** 2026-05-12

**Authors:** Akihiro Ishikawa, Masazumi Waseda, Tomoko Ishii, Yohei Kawai, Shin Kaneko

**Affiliations:** 1 Shin Kaneko Laboratory, Department of Clinical Application, Center for iPS Cell Research and Application (CiRA), Kyoto University, Kyoto 606-8507, Japan; 2 Laboratory of Cancer Immunology and Immunotherapy, Transborder Medical Research Center, University of Tsukuba, Tsukuba 305-8577, Japan

**Keywords:** cancer immunotherapy, tumor microenvironment, memory T cell, iPSC-derived CD8 T cell, l, iPSC-derived CAR-T cell, CXCR3, interleukin-15, JAK-STAT pathway, interleukin-21

## Abstract

The development of effective cellular immunotherapies for solid tumors requires the presence of robust infiltration, persistence, proliferation, and antigen-specific cytotoxicity. Here, we engineered induced pluripotent stem cell (iPSC)-derived cytotoxic T cells transduced with a chimeric antigen receptor (iCAR-T cells) and identified an optimal cytokine armoring strategy. Co-expression of interleukin-15 (IL-15) and IL-21 synergistically enhanced STAT1 phosphorylation, leading to increased transcriptional activation of the chemokine receptor CXCR3 and thereby improving tumor homing capacity. Furthermore, the engineered iCAR-T cells maintained a CD45RA^−^CD45RO^+^CCR7^+^CD62L^+^ memory T cell-like phenotype in tumors, contributing to the prolonged survival of the animal model. These findings demonstrate that cytokine synergy can be engineered into iPSC-derived T cells to overcome significant barriers in solid tumor immunotherapy, offering a scalable approach to developing next-generation off-the-shelf CAR-T therapies.

## Graphical abstract

**Figure:**
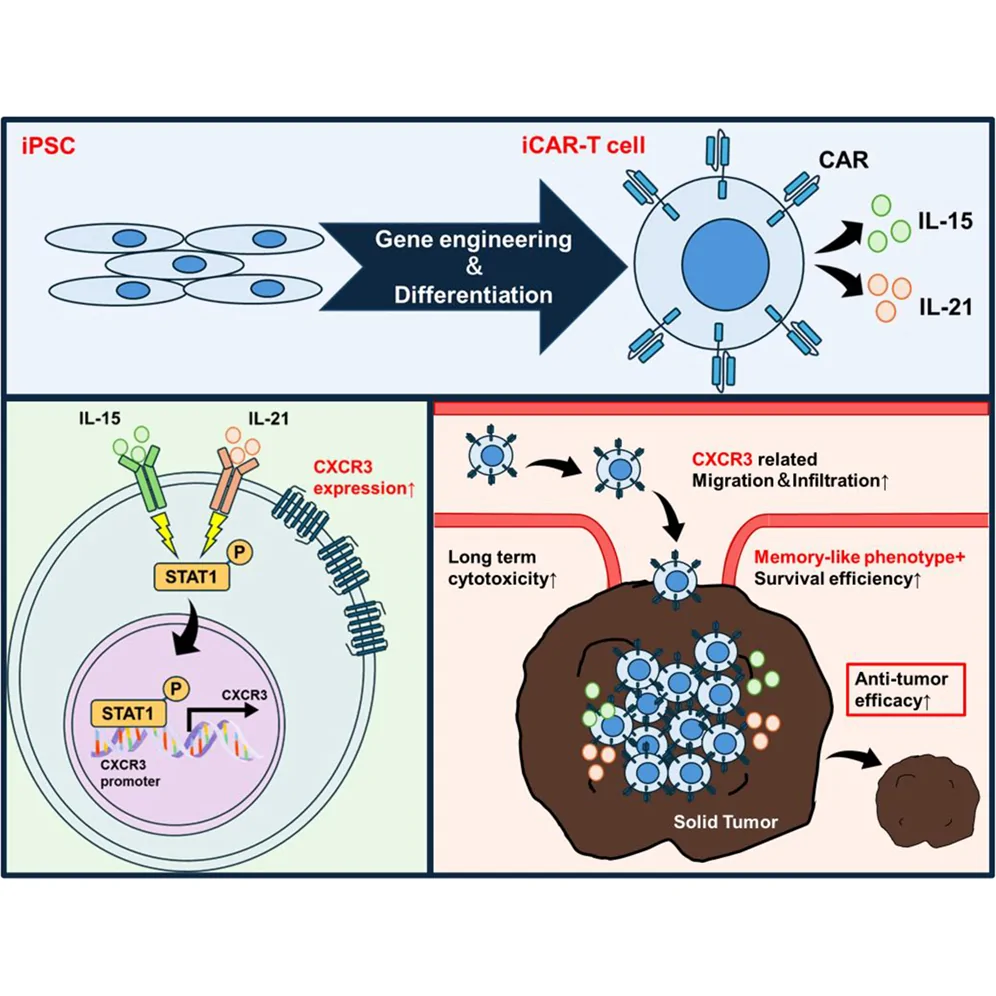

## Teaser

Standardizing solid-tumor CAR-T therapy is difficult. Kaneko and colleagues developed iPSC-derived CAR-T cells co-expressing IL-15 and IL-21, which synergistically drive STAT1-dependent CXCR3 migration and memory-like differentiation within tumors. This platform bridges the gap with primary T cells, providing a scalable, off-the-shelf strategy to overcome the suppressive tumor microenvironment.

## Introduction

Several studies have explored the elimination of cancer cells by activating the patient’s immune system, especially by activating T cells, which directly recognize and eliminate cancer cells.^1^^,^^2^^,^^3^ However, the number of T cells that recognize cancer cells in patients are naturally low and functionally insufficient due to the continuous exposure of the cells to cancer antigens for long periods.^4^ To obtain a large number of efficient T cells, the expression of chimeric antigen receptors (CARs) that specifically recognize target antigens on patient T cells has been widely studied and shown to have encouraging clinical outcomes for hematologic malignancies.^5^^,^^6^^,^^7^^,^^8^ However, less success has been achieved with solid tumors.^9^

To improve the anti-solid tumor activity, in general, T cells must continuously undergo a cancer immunity cycle in which they migrate to sites of the solid tumor, maintain proliferation, and express cytotoxicity.^10^^,^^11^^,^^12^ In the cancer immunity cycle, cytokines, such as interleukins (ILs) and chemokines, play important roles.^13^^,^^14^^,^^15^^,^^16^ ILs bind to receptors expressed on the T cell surface and enhance T cell functions through the JAK-STAT pathway.^16^ Cytokines that bind to the CD122 gamma-chain (γc) receptor, such as IL-2, IL-7, IL-15, and IL-21, and pro-inflammatory cytokines produced by innate immune cells, such as IL-12 and IL-18, contribute to T cell cytotoxicity and proliferation.^13^^,^^16^^,^^17^^,^^18^ Chemokines have been shown to play a major role through chemokine receptors for T cell migration to the tumor site.^12^^,^^14^^,^^15^ Tumor-infiltrating T cells have been reported to significantly express chemokine receptors, such as CXCR3 and CCR5, and migrate to tumor sites in response to several chemokines, such as CCL3, CCL4, and CCL5, and CXCL9, CXCL10, and CXCL11, which are secreted by cells in the tumor upon stimulation with interferon (IFN)-γ or other agents associated with inflammation.^12^^,^^19^ Additionally, several studies have reported armored CAR-T cells, in which CAR, ILs, and chemokines are introduced into T cells to improve therapeutic efficacy.^12^

Induced pluripotent stem cells (iPSCs) have attracted attention as a stable source for off-the-shelf regenerative medicine because of the ease of their genetic manipulation and evaluation of their safety as clonal cells.^20^ We have reported on CD8αβ^+^ T cells differentiated from iPSCs and their functionality.^21^^,^^22^^,^^23^ iPSCs can generate many functional T cells (iPS-T cells) due to their high proliferative potential and ease of multiplex gene editing as well as minimize inter-donor variability, giving them promise as a platform for the widespread use of T cell therapies.^20^^,^^24^ To achieve cancer immunotherapy using iPS-T cells, we reported that IL-15 enhances the function of CAR-expressing iPS-T cells (iCAR-T cells) and improves the anti-tumor efficacy against CD19^+^ B cell malignancy comparably with CAR-T cells.^24^ However, the efficacy was weak against solid tumors in an animal model; therefore, we considered searching for a cocktail of cytokines to activate iCAR-T cells.

We previously reported that the cytokine cocktail of IL-12, IL-15, IL-18, and IL-21 promotes the strong *in vitro* proliferation and effector function of iPS-T cells.^25^ Accordingly, we hypothesized that sustained cytokine cocktail signaling is important for maintaining iPS-T cell function locally in solid tumors. In the present paper, we show the enhanced anti-solid tumor effect of iCAR-T cells by an optimized cytokine combination that includes IL-15 and IL-21. IL-15 and IL-21 not only supported T cell survival and maintained a younger iCAR-T cell memory phenotype but also synergistically enhanced CXCR3-dependent migration ability through previously unreported signaling pathways. In summary, this study describes the mechanism responsible for improving the migration, proliferation, and memory phenotype of iCAR-T cells in solid tumors.

## Results

### IL-15-based cytokine combination improves iCAR-T cell cytotoxicity, proliferation, and anti-apoptotic activity

**Figure 1:**
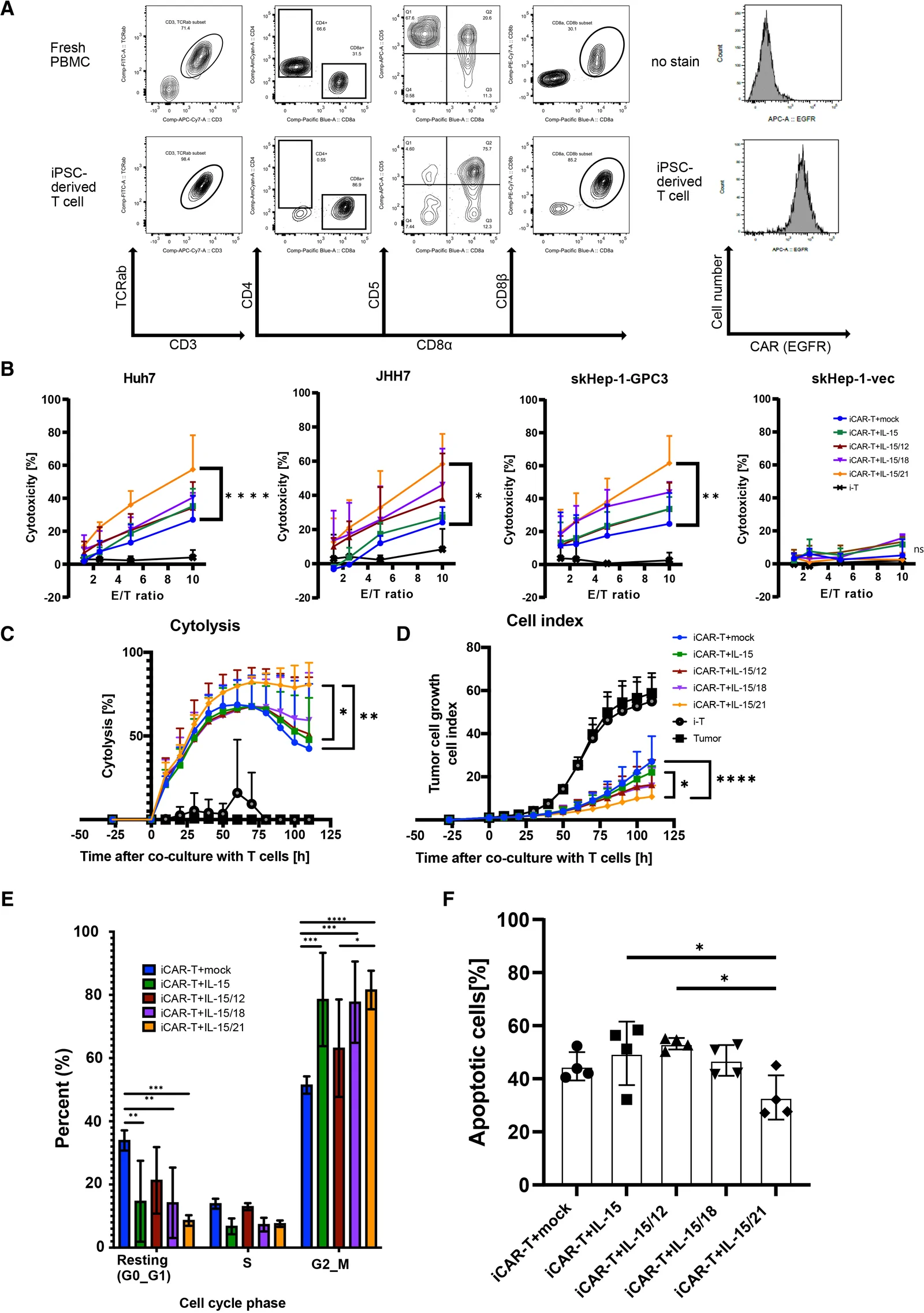
IL-15-based cytokine combination improved iCAR-T cell cytotoxicity, proliferation, and anti-apoptotic activity (A) Flow cytometry plots of iCAR-T cells after maturation. (B) Cytotoxicity of iCAR-T cells against Huh7, JHH7, skHep-1-GPC3, and skHep-1-vec cells. (C and D) Cytolysis and cell proliferation in response to skHep-1-GPC3 cells. (E) Representative cell-cycle progression of iCAR-T cells. (F) Representative percentage of annexin V and PI^+^ cells. Data represent the mean ± SEM of *n*-independent experiments. ∗*p* < 0.05; ∗∗*p* < 0.01; ∗∗∗*p* < 0.005; ∗∗∗∗*p* < 0.001; one-way or two-way ANOVA with Tukey’s multiple comparison test.

To investigate which cytokines contribute to the enhanced function of iPS-T cells, third-generation GPC3-specific CAR was transduced into iPSCs by a lentiviral vector. The CAR-modified iPSCs were differentiated to hematopoietic stem cells (HSCs) using the embryoid body (EB) method and then to CD4/8 double-positive T cells according to previous reports (Figures S1A–S1E).^23^^,^^24^ After a T cell receptor (TCR) stimulation-based maturation process, we obtained CD8αβ single-positive T cells expressing anti-GPC3-CAR (Figure 1A).^23^^,^^24^^,^^25^

We previously reported that recombinant human (rh)IL-12, rhIL-15, rhIL-18, and rhIL-21 significantly enhance the proliferation of iPS-T cells.^25^ To evaluate the impact on the proliferation of iCAR-T cells, rhIL-12, rhIL-15, rhIL-18, and rhIL-21 were added to the medium of a co-culture of iCAR-T cells and JHH7 cells expressing GPC3. rhIL-15 had the largest effect on proliferation based on accelerated cell cycling (Figures S1F and S1G). Based on these results, we combined each of the cytokines IL-12, IL-18, and IL-21 with IL-15 to stimulate iCAR-T cells in the following experiments (Figures S2A and S2B). To evaluate iCAR-T cell cytotoxicity, killing assays were performed. In short killing assays, all modified iCAR-T cells showed GPC3 antigen-mediated cytotoxicity against GPC3^+^ cancer cells (JHH7, Huh7, and skHep-1-GPC3) in a manner dependent on the effector:target (E:T) ratio (Figure 1B). In particular, iCAR-T cells expressing IL-15/IL-21 exhibited significantly higher cytotoxic activity compared with mock cells. In long-term killing assays, for which we used the xCelligence real-time cell analysis system, iCAR-T cells expressing IL-15/IL-21 maintained their cytotoxicity for more than 70 h and suppressed skHep-1-GPC3 cell growth; however, other iCAR-T cells did not show this effect (Figures 1C and 1D). Next, we examined the cytokine production, cell number, exhaustion, cell cycle, and apoptosis of the modified cells in co-culture with skHep-1-GPC3 cells to evaluate the function of each iCAR-T cell type relative to cytotoxicity. All modified iCAR-T cell types significantly increased their production of IL-2 and proinflammatory cytokines, such as IFN-γ and tumor necrosis factor (TNF), in the presence of the target antigen GPC3, except iCAR-T cells expressing IL-15/IL-12, which showed high production of IFN-γ even in the absence of GPC3 compared with other iCAR-T cells (Figure S2C). The antigen-driven proliferation revealed a significant contribution by all cytokine combinations except IL-15/IL-12 at day 7 from the initial CAR stimulation (Figure S2D). To understand the reason for the poor proliferation of iCAR-T cells expressing IL-15/IL-12, we evaluated the expression of the immune checkpoint molecules PD-1, LAG-3, and TIM-3 and found a significant increase in PD-1 and LAG-3 expression compared with other iCAR-T cell types (Figure S2E).

In general, T cell proliferation is strongly affected by cell-cycle progression and anti-apoptotic activity.^26^ To evaluate the cell-cycle status, each cytokine-expressing iCAR-T cell type was co-cultured with skHep-1-GPC3 cells for 3 days. iCAR-T cells expressing IL-15 only, IL-15/IL-18, or IL-15/IL-21 had a lower percentage of G0/G1 phase cells and a higher percentage of G2/M phase cells compared with mock cells; however, iCAR-T cells expressing IL-15/IL-12 showed no significant increase in dividing cells (Figures 1E and S2F). To evaluate the anti-apoptotic activity of each cytokine-expressing iCAR-T cell type, we checked the expression of annexin V, which is an early apoptosis marker. Fewer iCAR-T cells expressing IL-15/IL-21 showed late apoptosis after co-culture with skHep-1-GPC3 cells compared with iCAR-T cells expressing IL-15 (Figures 1F and S2G).

In summary, while several cytokine combinations enhanced effector functions, IL-15/IL-12 increased iCAR-T cell immune checkpoint expression and reduced proliferation, IL-15/IL-18 enhanced the function of iCAR-T cells *in vitro*, and IL-15/IL-21 enhanced long-term killing.

### iCAR-T cells expressing IL-15/IL-21 suppress tumor progression and prolong survival of solid tumor animal models

**Figure 2:**
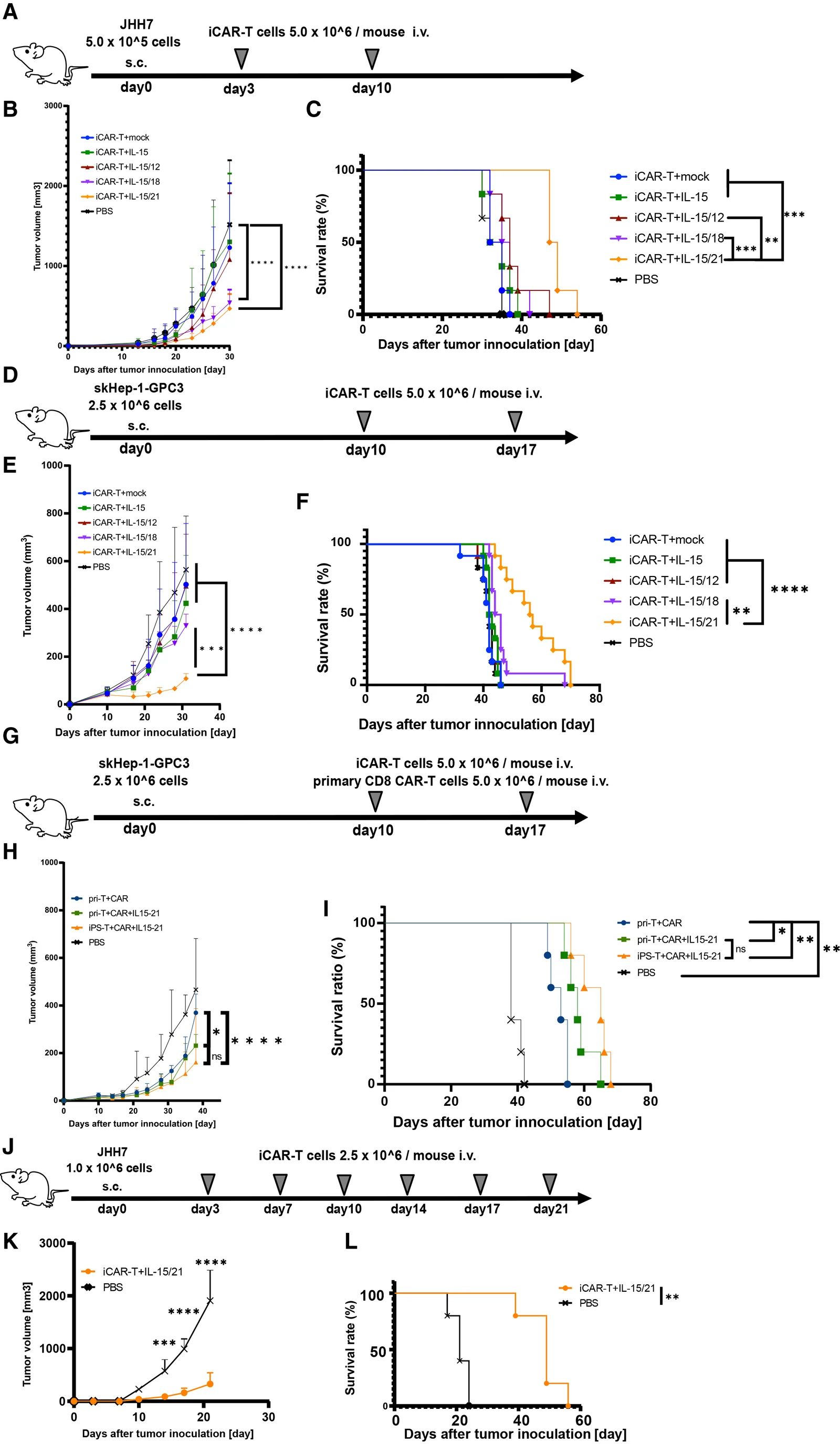
iCAR-T cells expressing IL-15/IL-21 suppress tumor volume progression and improve mouse survival in a subcutaneous tumor model (A) Schedule of the JHH7 cell inoculation and iCAR-T cell injection. 5.0 × 10 JHH7 cells were subcutaneously injected into the left back side of mice. (B and C) Tumor volume and mouse survival rate after injection of each iCAR-T cell type (*n* = 6 mice each). (D) Schedule of the skHep-1-GPC3 cell inoculation and iCAR-T cell injection. We subcutaneously injected 2.5 × 10 skHep-1-GPC3 cells into the left back side of the mice. (E and F) Tumor volume and mouse survival rate after injection of each iCAR-T cell type (*n* = 12 mice each). (G) Schedule of the skHep-1-GPC3 cell inoculation and iCAR-T cell injection. We subcutaneously injected 2.5 × 10 skHep-1-GPC3 cells into the left back side of the mice. (H and I) Tumor volume and mouse survival rate after injection of each iCAR-T cell type (*n* = 4 mice each). (J) Schedule of the JHH7 cell inoculation and iCAR-T cell expressing IL-15/IL-21 injection. We subcutaneously injected 1.0 × 10 JHH7 cells into the left back side of the mice. (K and L) Tumor volume and mouse survival rate after injection of each iCAR-T cell type (*n* = 5 mice each). ∗*p* < 0.05; ∗∗*p* < 0.01; ∗∗∗*p* < 0.005; ∗∗∗∗*p* < 0.001; one-way ANOVA with Tukey’s multiple comparison test.

To observe the therapeutic efficacy of iCAR-T cells, JHH7 or skHep-1-GPC3 cells were subcutaneously inoculated into NOD-SCID IL2Rγc^null^ (NSG) mice, and one modified iCAR-T cell type was subsequently injected intravenously (Figures 2A, 2D, 2G, and 2J). For both animal models (JHH7 and skHep-1-GPC3 cell inoculation), tail vein injection of iCAR-T cells expressing IL-15/IL-21 significantly suppressed the tumor progression and significantly prolonged the survival of both animal models compared with other iCAR-T cells (Figures 2B, 2C, 2E, and 2F). To evaluate the potential of iPS-T cells expressing IL-15/IL-21 as a candidate off-the-shelf allogeneic CD8 T cell platform for CAR modification, we compared healthy volunteer-derived GPC3-specific CAR-transduced CD8 CAR-T (primary CD8 CAR-T) cells expressing IL-15/IL-21.^27^ Primary CD8 CAR-T cells and those expressing IL-15/IL-21 were made and evaluated for their cell surface markers, cytotoxicity, and cytokine production. IL-15/IL-21-expressing iCAR-T cells possessed cell surface markers similar to those of primary CD8 CAR-T cells (Figure S3A). Antigen-specific activity verified that iCAR-T cells expressing IL-15/IL-21 and primary CD8 CAR-T cells exhibited specific cytotoxic activity against the GPC3 antigen and significantly expressed IFN-γ, IL-2, and TNF (Figures S3B and S3C). Finally, the anti-tumor efficacy against solid tumors was confirmed *in vivo* using the same conditions as in Figures 2D and 2G. Both iCAR-T cells and primary CAR-T cells expressing IL-15/IL-21 similarly suppressed tumor progression more effectively than primary CD8 CAR-T cells without the cytokine modification (Figure 2H). In addition, the survival rate of mice treated with both types of cytokines was significantly enhanced (Figure 2I). One advantage of using iPSC-derived cells for clinical applications is the ability to administer multiple doses to treat a patient. From this perspective, we evaluated the therapeutic efficacy of iCAR-T cells expressing IL-15/IL-21 in a repeated administration setting, as shown in Figure 2J. Compared to the experiment in Figure 2A, the number of inoculated tumor cells was twice as high, the number of cells per injection was half, and injections were three times more frequent (administered six times). This treatment resulted in significantly prolonged survival of the animal model with higher tumor inoculation (Figures 2K and 2L).

In summary, iCAR-T cells expressing IL-15/IL-21 suppressed the progression of solid tumors and improved survival better than the other iCAR-T cells.

### iCAR-T cells expressing IL-15/IL-21 had improved persistency in a solid tumor animal model

**Figure 3:**
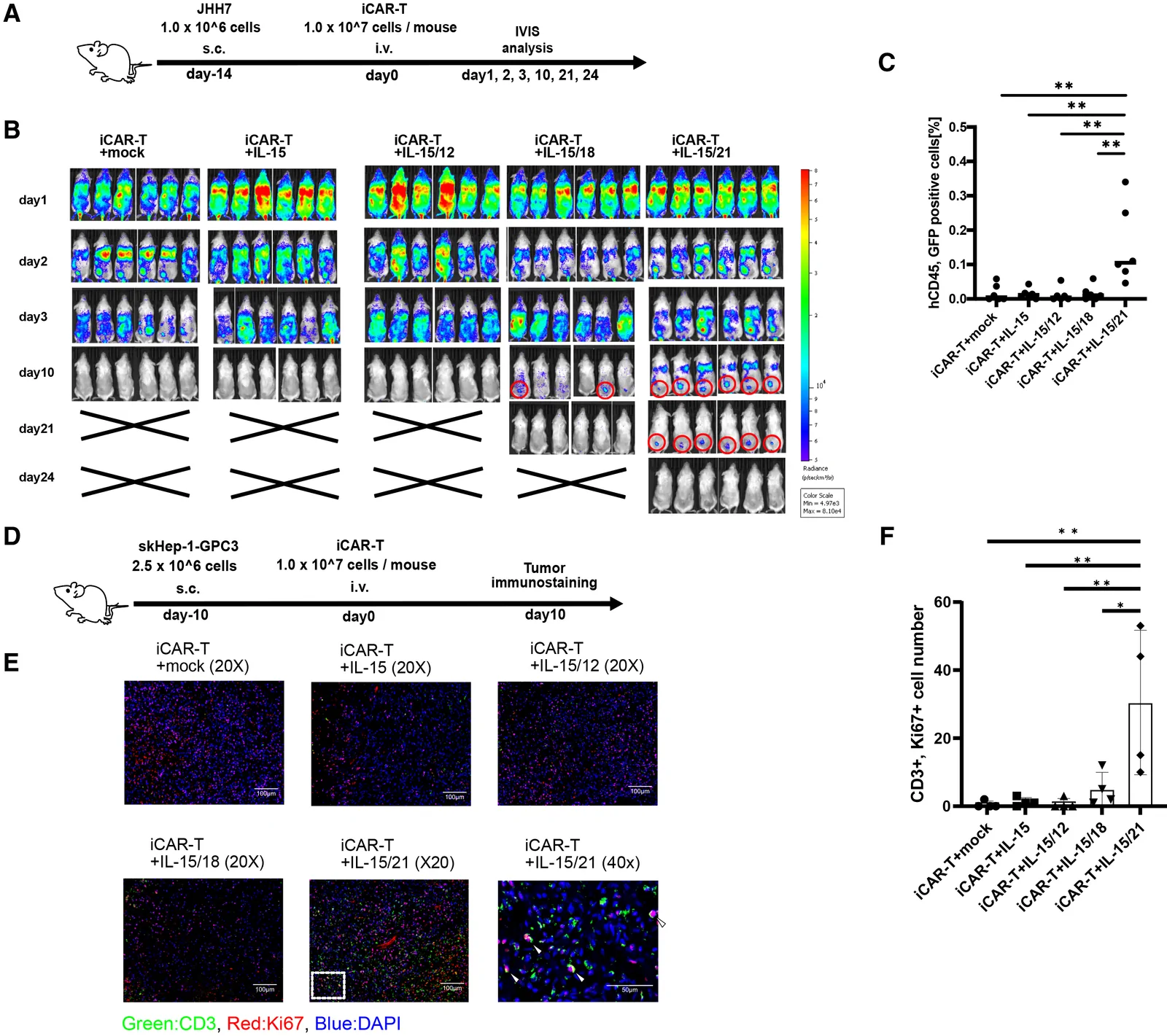
iCAR-T cells expressing IL-15/IL-21 have enhanced survival in tumors (A) Schedule of the JHH7 cell inoculation and iCAR-T cell injection. We subcutaneously injected 1.0 × 10 JHH7 cells into the left back side of the mice. (B) Luciferase luminescence around the tumor (*n* = 6). Luciferase was expressed by iCAR-T cells. Red circles show luciferase luminescence around the tumor. (C) hCD45^+^ GFP^+^ cell population in mouse peripheral blood 7 days after the injection. (D) Schedule of the skHep-1-GPC3 cell inoculation and iCAR-T cell injection. We subcutaneously injected 2.5 × 10 skHep-1-GPC3 cells into the left back side of the mice. (E) Immunofluorescence staining of skHep-1-GPC3 cells derived from subcutaneous carcinoma in mice treated with iCAR-T cells (green: human CD3, red: Ki67, blue: DAPI). Arrowheads indicate human CD3^+^Ki67^+^ cells. (F) Statistical analysis of infiltrated CD3^+^ and Ki67^+^ iCAR-T cells. ∗*p* < 0.05; ∗∗*p* < 0.01; one-way ANOVA with Tukey’s multiple comparison test.

The enhancement of T cell survival efficacy and proliferation *in vivo* is important for anti-tumor efficacy.^10^^,^^28^^,^^29^^,^^30^ We therefore examined the survival efficacy of iCAR-T cells that were genetically modified by luciferase and GFP *in vivo*. To evaluate iCAR-T cells in tumors, *in vivo* imaging was done under the schedule shown in Figure 3A. The luminescence intensity around the tumors was maintained in mice injected with iCAR-T cells expressing IL-15/IL-21 compared with other iCAR-T cell groups (Figure 3B). Additionally, only iCAR-T cells expressing IL-15/IL-21 were detected in peripheral blood (PB) 7 days after injection (Figure 3C). To confirm iCAR-T cells in tumors, the tumors were stained with anti-human CD3 and anti-human Ki67 antibodies; Ki67 is a proliferation-related protein (Figure 3D). The subcutaneous tumors of mice treated with iCAR-T cells expressing IL-15/IL-21 showed more human CD3^+^Ki67^+^ cells than other iCAR-T cells (Figures 3E and 3F). Finally, we observed 34 standard cell surface markers on tumor-infiltrating iCAR-T cells by cytometry by time of flight (CyTOF) mass spectrometry (Figure S4A). iCAR-T cells expressing IL-15/IL-21 formed a separate cluster and unique expression of memory-related cell surface markers such as CD45RA, CD45RO, CD62L, CCR7, and CXCR3 compared with other iCAR-T cells (Figure S4B).

In summary, iCAR-T cells expressing IL-15/IL-21 showed longer persistence and better proliferation in tumors compared with other iCAR-T cells. In addition, tumor-infiltrating iCAR-T cells expressing IL-15/IL-21 showed a unique expression of CD45RA, CD45RO, CD62L, CCR7, and CXCR3 compared with other iCAR-T cells.

### iCAR-T cells expressing IL-15/IL-21 have enhanced CXCR3 expression, which increased the migration ability to solid tumors

**Figure 4:**
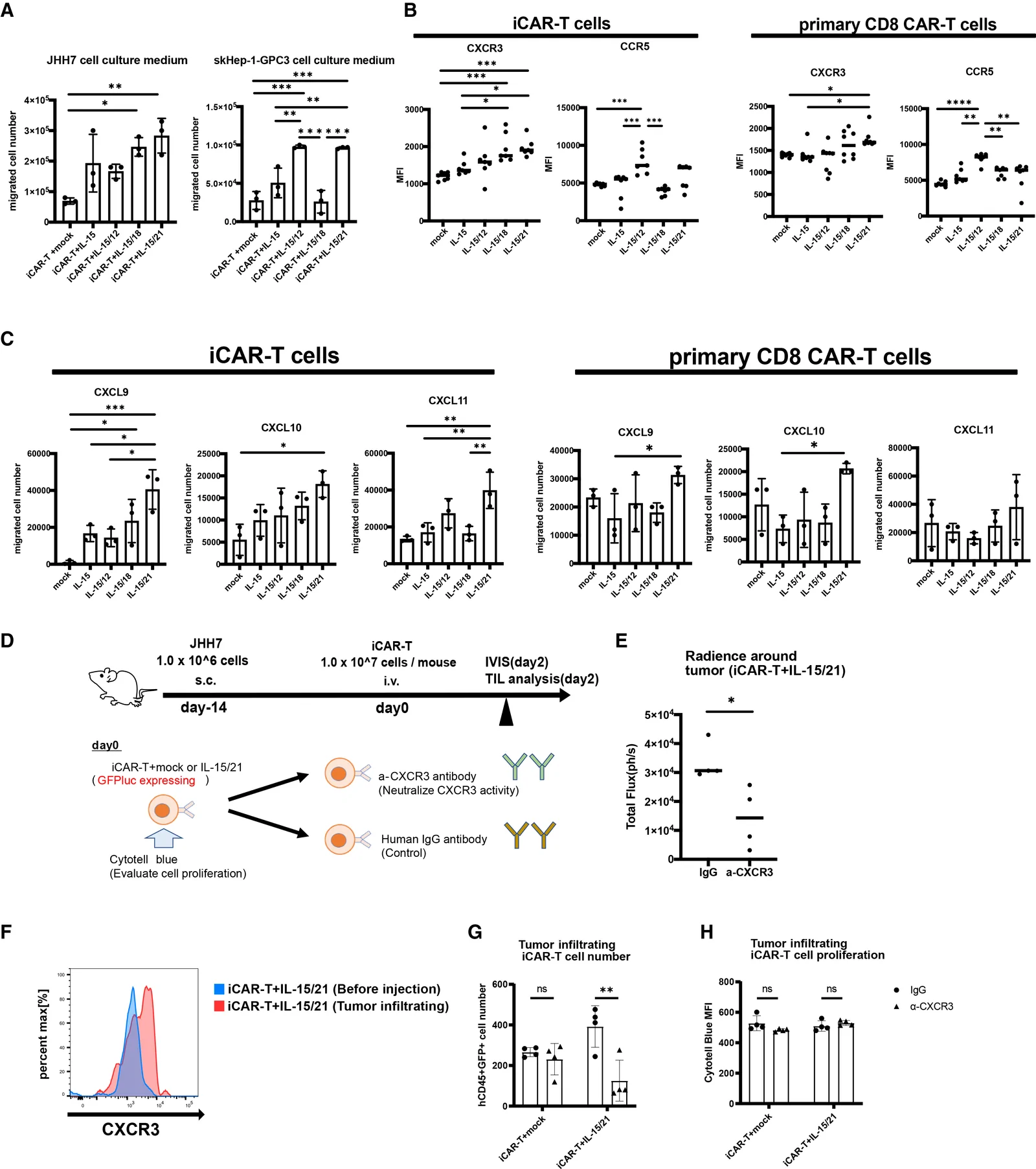
iCAR-T cells expressing IL-15/IL-21 show enhanced CXCR3 related migration (A) Migration activity of iCAR-T cells against JHH7 and skHep-1-GPC3 cell culture medium (*n* = 3). (B) CXCR3 and CCR5 expression on iCAR-T and primary CD8 CAR-T cells (*n* = 7). (C) Migration activity against CXCL9, CXCL10, and CXCL11 by iCAR-T and primary CD8 CAR-T cells (*n* = 3). (D) Scheme of the migration assay *in vivo*. We subcutaneously injected 1.0 × 10 JHH7 cells in the left back side of the mice. (E) Luciferase luminescence around the tumor 2 days after injection of iCAR-T cells expressing IL-15/IL-21 (*n* = 4). (F) Flow cytometry plots of CXCR3 expression on iCAR-T cells expressing IL-15/IL-21 in the tumor and infused with human IgG antibody. (G) hCD45^+^lucGFP^+^ cell number in the tumor on day 2 (*n* = 4). (H) CytoTell Blue expression in iCAR-T cells expressing IL-15/IL-21 in the tumor on day 2 (*n* = 4). ∗*p* < 0.05; ∗∗*p* < 0.01; ∗∗∗*p* < 0.005; ∗∗∗∗*p* < 0.001; one-way ANOVA with Tukey’s multiple comparison test and Student’s *t* test.

To start and accelerate the cancer-immunity cycle, cytotoxic CD8 T cells exert anti-tumor effector functions after migrating to the tumor sites.^10^ Therefore, we evaluated the migration activity of iCAR-T cells *in vitro* by counting the number of migrating iCAR-T cells passing transwell membranes toward the cultured supernatant of JHH7 or skHep-1-GPC3 cells. In the assay, all modified iCAR-T cells commonly showed more migrating cells; however, the biggest effect was associated with the IL-15/IL-21 modification (Figure 4A). We speculated the reason was higher chemokine sensitivity; therefore, we evaluated the expression of CXCR3 and CCR5 on iCAR-T cells, as they are representative chemokine receptors that are highly expressed on tumor-infiltrating CD8-T cells.^15^ Using flow cytometry, we detected significantly enhanced CXCR3 expression on iCAR-T cells expressing IL-15/IL-18 or IL-15/IL-21 and primary CD8 CAR-T cells expressing IL-15/IL-21 (Figure 4B). In contrast, the IL-15/IL-12 modification led to more CCR5 expression (Figure 4B). iCAR-T cells expressing IL-15/IL-21 exhibited significantly superior migration activity toward the CXCR3 ligands CXCL9, CXCL10, and CXCL11 compared with iCAR-T cells expressing IL-15 only or mock (Figure 4C, left).^31^^,^^32^ A comparable tendency has also been observed in primary CD8 CAR-T cells (Figure 4C, right). Consistently, neutralizing the CXCR3 activity of iCAR-T cells using anti-CXCR3 antibody significantly inhibited the migration ability against supernatants of JHH7 or skHep-1-GPC3 cells and CXCL11, the ligand of CXCR3 (Figures S5A and S5B). Finally, to evaluate differences in iCAR-T cells expressing IL-15/IL-18 or IL-15/IL-21 with enhanced CXCR3 expression, we measured the number of CXCR3-related migrating cells with different chemokine ligand concentrations. iCAR-T cells expressing IL-15/IL-21 migrated even at low concentrations of chemokine ligands compared with iCAR-T cells expressing IL-15/IL-18 (Figure S5C).

Next, we evaluated the correlation of CXCR3 expression in iCAR-T cells expressing IL-15/IL-21 and migration to the tumor. For the experiments, these cells were modified to express luciferase and stained using CytoTell Blue cell dye to separate cell proliferation from chemotaxis. iCAR-T cells were treated with anti-CXCR3 neutralizing antibody or isotype human immunoglobulin G (IgG) antibody and then injected intravenously into NSG mice that had been subcutaneously inoculated with JHH7 cells, which highly express CXCL9, CXCL10, and CXCL11 (Figures 4D and S5D). A luminescence-based *in vivo* imaging system was used to evaluate the iCAR-T cell accumulation at the tumor. At 48 h after the injection of iCAR-T cells expressing IL-15/IL-21, the anti-CXCR3 antibody-treated group showed significantly less radiance around the tumor compared with the IgG-treated group (Figure 4E). Additionally, we evaluated the CXCR3 expression on and number of tumor-infiltrating iCAR-T cells. CXCR3 expression was enhanced in cells treated with human IgG antibody (Figure 4F). We evaluated the tumor-infiltrating hCD45^+^lucGFP^+^ cell number and found it was significantly less among iCAR-T cells expressing IL-15/IL-21 treated with anti-CXCR3 antibody (Figure 4G). However, there was no significant difference in the mean fluorescence intensity of CytoTell Blue between the anti-CXCR3-and IgG-treated groups (Figure 4H).

These data indicate that the increased number of tumor-infiltrating iCAR-T cells expressing IL-15/IL-21 48 h after the injection was due to accelerated CXCR3-mediated homing to the tumor and not due to proliferation at the tumor site.

### IL-15 and IL-21 synergistically enhance phosphorylation of STAT1, which directly binds to the CXCR3 promoter to enhance its expression

**Figure 5:**
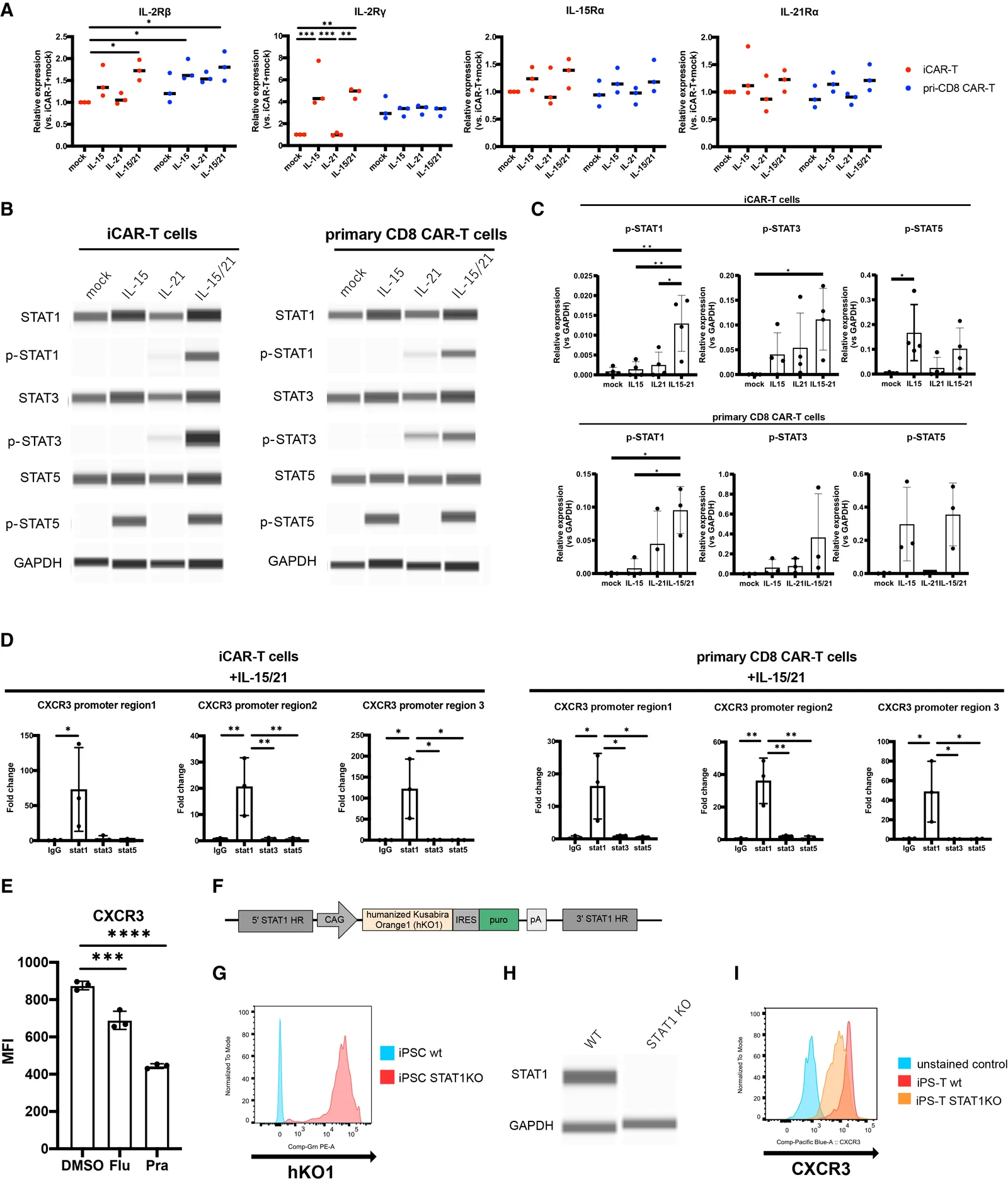
iCAR-T cells expressing IL-15/IL-21 show enhanced CXCR3 expression via STAT1 signaling (A) Expression of IL-2Rβ, IL-2Rγ, IL-15Rα, and IL-21Rα on iCAR-T and primary CD8 CAR-T cells. (B and C) Protein expression and phosphorylation of STAT1, STAT3, and STAT5 proteins on iCAR-T and primary CD8 CAR-T cells (*n* = 4). (D) ChIP-qPCR results of iCAR-T and primary CD8 CAR-T cells expressing IL-15/IL-21 cells with anti-STAT1, anti-STAT3, and anti-STAT5 antibodies (*n* = 3). (E) CXCR3 expression in iCAR-T cells expressing IL-15/IL-21 treated with 100 μM DMSO, fludarabine (Flu), or pravastatin (Pra). (F) Schematic of the STAT1-KO vector. (G) Flow cytometry plot of humanized Kusabira Orange1 (hKO1) expression after puromycin treatment. (H) Protein expression of STAT1 in wild-type (WT) and STAT1-KO iPSC clones. (I) CXCR3 expression on iPS-T cells. ∗*p* < 0.05; ∗∗*p* < 0.01; ∗∗∗*p* < 0.005; ∗∗∗∗*p* < 0.001; one-way ANOVA with Tukey’s multiple comparison test.

We next investigated how CXCR3 expression on iCAR-T cells is regulated by the expression of IL-15 and IL-21. IL-2Rγc binding cytokines, such as IL-2, IL-7, IL-15, and IL-21, are known to phosphorylate the tyrosine residues of STAT1, STAT3, and STAT5 through the phosphorylation of JAKs to generate the active form of STATs, which can then bind to multiple gene promotors as transcription factors.^33^ We evaluated cytokine receptors expression related to IL-15 and IL-21 and found that IL-2Rγ expression was significantly enhanced on iCAR-T cells expressing IL-15/IL-21 compared with mock cells but not in primary CD8 CAR-T cells (Figure 5A). Next, we evaluated the amount of phosphorylated (p-)STAT1, p-STAT3, and p-STAT5 on iCAR-T cells genetically modified to express IL-15, IL-21, or both. As with PB-derived CD8 T cells, IL-15- and IL-21-mediated signal enhanced the phosphorylation of STAT1, STAT3, and STAT5 on iCAR-T cells (Figures 5B, 5C, and S6A). Based on the synergistic phosphorylation of STAT1 upon IL-15 and IL-21 activation, we hypothesized that p-STAT1, but neither p-STAT3 nor p-STAT5, regulates CXCR3 expression. To evaluate chromatin accessibility around CXCR3, we first performed assay for transposase accessible chromatin sequencing (ATAC-seq), finding that the upstream regions of CXCR3 were open in iCAR-T cells and primary CD8 CAR-T cells (Figure S6B). We used a JASPAR analysis to investigate which transcription factors bind upstream of the CXCR3 chromatin open regions and found STAT1 to be a candidate (Figure S6C). Next, we performed chromatin immunoprecipitation-quantitative PCR (ChIP-qPCR) with anti-STAT1, anti-STAT3, and anti-STAT5 antibodies on three different promoter regions of CXCR3 to determine the direct involvement of STATs in the transcription of CXCR3. Using three different primer pairs within the CXCR3 promoter, we found p-STAT1 significantly bound to multiple target regions/sequences in CXCR3 promoter in iCAR-T and primary CD8 CAR-T cells expressing IL-15/IL-21 (Figure 5D). To confirm the contribution of p-STAT1 to CXCR3 expression, we inhibited the phosphorylation using STAT1 inhibitors. These inhibitors significantly decreased the expression of CXCR3 on iCAR-T cells expressing IL-15/IL-21 (Figure 5E). In addition, we prepared STAT1-knockout (KO) iCAR-T cells using CRISPR-Cas9 to further estimate the STAT1 effect on CXCR3 (Figure 5F). We found that CXCR3 expression was decreased on STAT1-KO iPS-T cells compared with wild-type cells (Figures 5G–5I). To evaluate the involvement of STAT3 and STAT5 in CXCR3 expression, short hairpin RNA (shRNA) was used. shRNA corresponding to STAT3 and STAT5 decreased the expression levels of STAT3 and STAT5 in iCAR-T cells as well as the proliferation capacity (Figures S7A and S7B). However, no difference in CXCR3 expression was observed under different shRNA conditions (Figure S7C).

Next, we evaluated the contribution of CXCR3 and STAT1 to the anti-tumor efficacy of iCAR-T cells expressing IL-15/IL-21. CXCR3 was knocked out using CRISPR-Cas9, and the resulting impact on proliferation capacity was evaluated (Figure S7D). CXCR3-KO did not affect proliferation capacity, but STAT1-KO reduced it compared with wild-type cells (Figure S7E). In addition, both KO cells exhibited reduced chemotaxis toward CXCL9, CXCL10, and CXCL11, and STAT1-KO cells showed significantly decreased IFN-γ and GZMB expression compared with wild-type cells (Figures S7F and S7G). Finally, using a mouse subcutaneous tumor model to evaluate the suppression of solid tumor growth, CXCR3-KO or STAT1-KO iCAR-T cells correlated with significantly less anti-tumor efficacy by iCAR-T cells expressing IL-15/IL-21 (Figures S7H–S7J).

In summary, IL-15 and IL-21 enhanced STAT1, STAT3, and STAT5 phosphorylation in both iCAR-T and primary CD8^+^ CAR-T cells, with a synergistic effect on STAT1 phosphorylation and CXCR3 expression. To establish a causal link with the IL-15/IL-21-STAT1--CXCR3 axis, we performed loss-of-function experiments. Combined IL-15/IL-21 signaling induced robust STAT1 phosphorylation, which in turn promoted CXCR3 expression. Notably, genetic ablation of either STAT1 or CXCR3 significantly impaired the migratory capacity of iCAR-T cells toward tumor sites. These findings demonstrate that STAT1 serves as a critical transcriptional mediator linking IL-15/IL-21 signaling to CXCR3-driven tumor infiltration, with CXCR3 acting as a key downstream effector of STAT1-dependent trafficking in iCAR-T cells expressing IL-15/IL-21.

### Tumor-infiltrating iCAR-T cells expressing IL-15/IL-21 showed memory-T cell like phenotype

**Figure 6:**
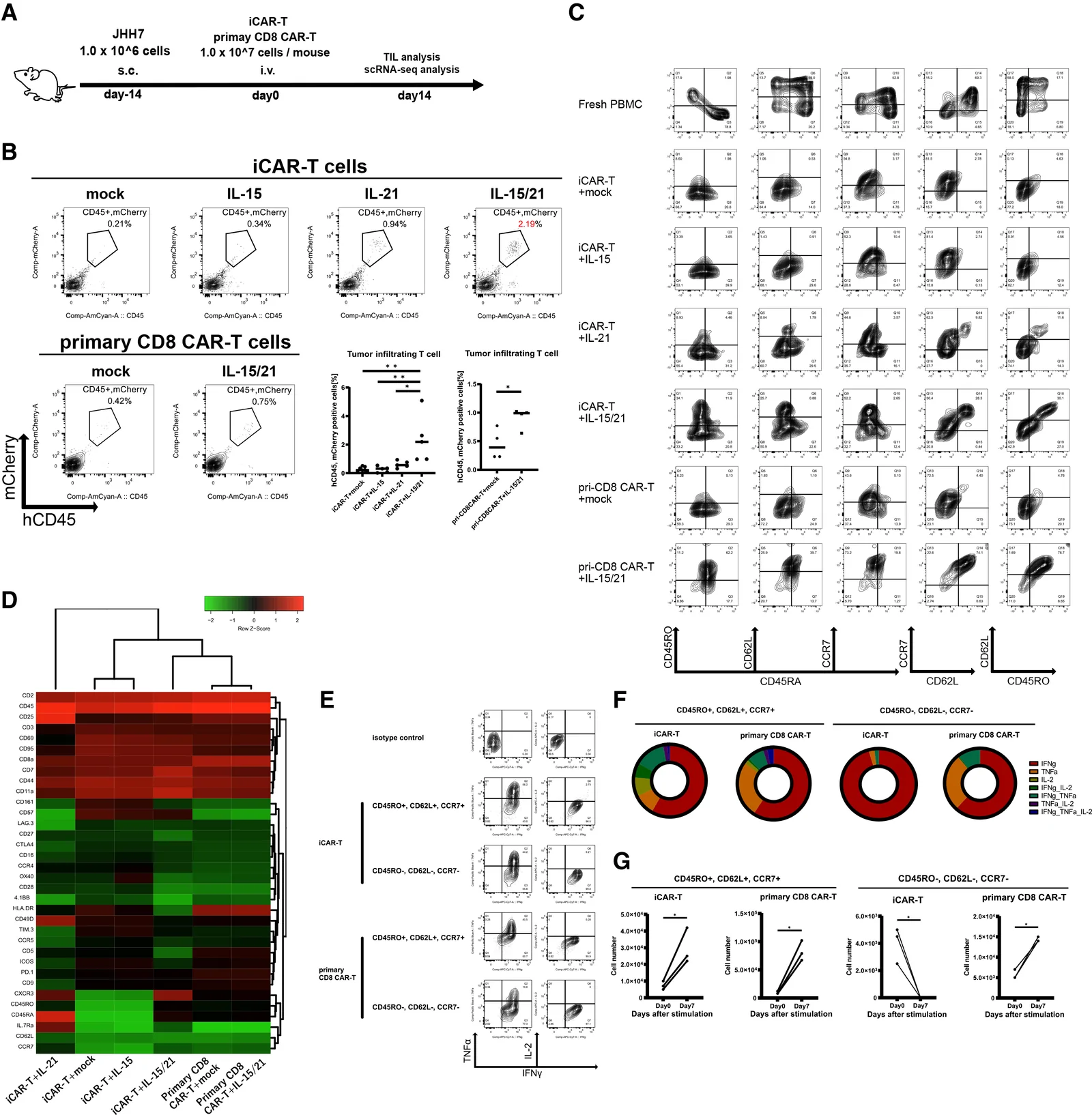
iCAR-T cells expressing IL-15/IL-21 showed memory phenotype in tumors (A) Schedule of the JHH7 cell inoculation and iCAR-T cell injection. We subcutaneously injected 1.0 × 10 JHH7 cells into the left back side of the mice. (B and C) Representative flow cytometry plots of iCAR-T and primary CD8 CAR-T cell populations and memory-related cell surface markers in tumors. (D) Cell surface gene expression heatmap of iCAR-T and primary CD8 CAR-T cells by CyTOF mass spectrometry. (E and F) IFN-γ, TNF-α, and IL-2 expression in tumor-infiltrating iCAR-T and primary CD8 CAR-T cells expressing or not expressing CD45RO, CD62L, and CCR7 stimulated with phorbol 12-myristate 13-acetate ionomycine. (G) Cell number of tumor-infiltrating iCAR-T and primary CD8 CAR-T cells expressing or not expressing CD45RO, CD62L, and CCR7 after stimulation with anti-human CD3 agonist antibody. ∗*p* < 0.05; ∗∗*p* < 0.01; one-way ANOVA with Tukey’s multiple comparison test and Student’s *t* test.

One favorable phenotype of therapeutic T cells in patients that relates to clinical efficacy against cancer is the early memory T cell phenotype.^34^ To evaluate the synergistic effect of IL-15 and IL-21 gene modification on the phenotype of iCAR-T cells in a subcutaneous tumor-bearing animal model, tumor-infiltrating T cells were analyzed 14 days after injection into tail vein (Figure 6A). iCAR-T cells expressing IL-15/IL-21 exhibited the best survival efficiency to the subcutaneous tumor compared with other iCAR-T cells (Figure 6B). Next, we evaluated each T cell phenotype in the tumor by flow cytometry and found that T cells expressing the central memory T cell markers CD45RA^neg^, CD45RO^pos^, CD62L^pos^, and CCR7^pos^ were retained in mice treated with IL-15 and IL-21 gene-modified iCAR-T cells or primary CD8 CAR-T cells (Figure 6C). CyTOF mass spectrometry showed that cell surface markers on iCAR-T and primary CD8 CAR-T cells expressing IL-15/IL-21 infiltrating the tumor were similar (Figure 6D). We also confirmed the production of multiple cytokines (IL-2, IFN-γ, and TNF-α) and the proliferative potential of CD45RO^+^, CD62L^+^, and CCR7^+^ iCAR-T cells but not in negative cells (Figures 6E–6G).

**Figure 7:**
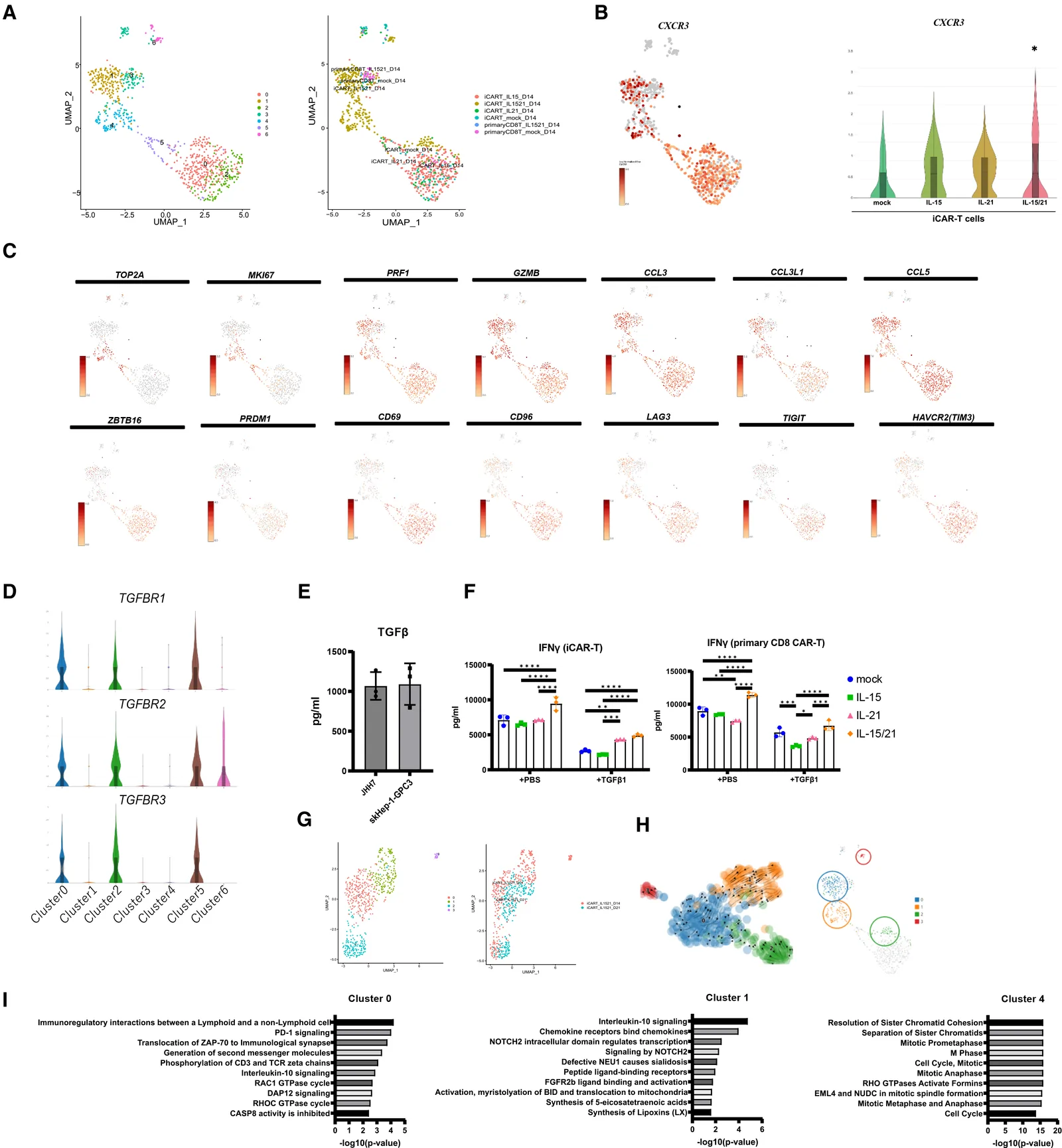
IL-15 and IL-21 synergistically affect iCAR-T cell phenotype in tumors (A) UMAP plots of tumor-infiltrating T cells. (B) Representative expression of CXCR3 in each sample shown as UMAP and violin plots. (C) Representative expression of TOP2A, MKI67, PRF1, GZMB, CCL3, CCL3L1, CCL5, ZBTB16, PRDM1, CD69, CD96, LAG3, TIGIT, and HAVCR2 (TIM3) in each cluster shown as UMAP plots. (D) Representative single-cell expression of TGFBR1, TGFBR2, and TGFBR3 in each sample shown as violin plots. (E) TGF-β expression of JHH7 and skHep-1-GPC3 cells. (F) IFN-γ expression of iCAR-T and primary CD8 CAR-T cells expressing each cytokine under TGF-β1 stimulation. (G) UMAP plots of day 14 and day 21 tumor-infiltrating iCAR-T cells expressing IL-15/IL-21. (H) scRNA-seq velocity analysis of iCAR-T cells expressing IL-15/IL-21. (I) A pathway and process enrichment analysis was performed on upregulated genes in clusters 0, 1, and 4. ∗*p* < 0.05; ∗∗*p* < 0.01; ∗∗∗*p* < 0.005; ∗∗∗∗*p* < 0.001; one-way ANOVA with Tukey’s multiple comparison test.

To further evaluate the phenotype of tumor-infiltrating T cells, we conducted single-cell RNA-seq (scRNA-seq) using tumor-infiltrating iCAR-T cells expressing mock, IL-15, IL-21, or IL-15/IL-21 and primary CD8 CAR-T cells expressing mock or IL-15/IL-21. We also performed a pathway analysis using genes whose expression varied significantly in each sample. We found that iCAR-T cells expressing IL-15/IL-21 showed an enhanced immune system and chemokine-related pathways compared with other iCAR-T cells (Table S1). Merged uniform manifold approximation and projection (UMAP) plots of those samples revealed seven distinct clusters (Figure 7A). The populations of iCAR-T cells expressing mock, IL-15, and IL-21 were mainly found in clusters 0, 2, and 5, while those of iCAR-T cells expressing IL-15/IL-21 were in clusters 0, 1, and 4. Primary CD8 CAR-T cells were found in cluster 3 and in the close neighborhood of clusters 1 and 4, where iCAR-T cells expressing IL-15/IL-21 were abundant. In contrast to the heterogeneous clusters of iCAR-T cells expressing IL-15/IL-21, the other iCAR-T cell populations were mainly in cluster 0. Additionally, we investigated genes significantly expressed by iCAR-T cells expressing IL-15/IL-21, finding that CXCR3 expression was significantly higher compared with other iCAR-T cells (Figure 7B). We observed genes significantly expressed in clusters where iCAR-T cells expressing IL-15/IL-21 were mainly located: cell cycle and cell-cycle progression-related genes such as TOP2A and MKI67 in cluster 4; effector granules, such as GZMB and PRF1, and chemokine ligands, such as CCL3, CCL3L1, and CCL5, in cluster 1; and ZBTB16, PRDM1, CD69, CD96, LAG3, TIGIT, and HAVCR2 (TIM3) in clusters 0 and 2 (Figure 7C). Next, we analyzed sensitivity to transforming growth factor β (TGF-β), which is frequently observed in the tumor microenvironment (TME). The results showed that the expression of TGF-β receptors was reduced in clusters 1 and 4, which is where iCAR-T cells expressing IL-15/IL-21 mostly existed (Figure 7D). Tumors used in our animal model, JHH7 and skHep-1-GPC3 cells, produced high levels of TGF-β and iCAR-T, and primary CD8-CAR-T cells expressing IL-15/IL-21 maintained IFN-γ expression at relatively high levels even under high concentrations of rhTGF-β1 compared with other iCAR-T cells (Figures 7E and 7F). scRNA-seq analysis performed 14 and 21 days after iCAR-T cell injection caused no unique clusters between the two days (Figure 7G). An RNA velocity analysis of the clusters including iCAR-T cells expressing IL-15/IL-21 revealed the universal kinetics of iCAR-T cells from cluster 4 to cluster 0 via cluster 1 (Figure 7H). Finally, clusters including iCAR-T cells expressing IL-15/IL-21 were characterized by Gene Ontology and pathway profiles. Cluster 4 expressed genes related to cell division, and cluster 1 expressed genes related to the chemokine/chemokine receptor. Cluster 0 expressed genes related to effector T cell phenotype such as TCR signaling and exhaustion (Figure 7I).

In summary, iCAR-T and primary CD8 CAR-T cells expressing IL-15/IL-21 expressed memory T cell-like markers, such as CD45RO, CD62L, and CCR7, as well as multiple cytokines and showed higher proliferation potential.

## Discussion

iPSC-derived antigen-specific receptor-expressing cytotoxic CD8 T cells were first reported in 2013; however, the therapeutic efficacy of these cells in tumor-bearing animal models was insufficient. Since then, various modifications to improve the quality of the cells have been pursued. It is reported that three signals are important for T cell activation: the first is mediated by TCR; the second by co-stimulatory molecules, such as CD28 and 4-1BB; and the third by cytokines.^12^^,^^35^^,^^36^ CAR plays a critical role in the first and second signals, but the resulting anti-tumor efficacy is insufficient against solid tumors.^9^ To enhance the efficacy, a third signal should be modified by the expression of cytokines or cytokine receptor genes.^35^^,^^36^ Among these genes, those that supply cytokine signals to human iPSC-derived CD8αβ killer T cells were found to improve persistence in an animal model. The intravenous injection of iPSC-derived CD8αβ killer T cells that were retrovirally modified with CD19 CAR and IL-15/IL-15Rα genes significantly prolonged the survival of NSG mice that were inoculated with a human B cell leukemia cell line, Nalm-6, as long as the mice were treated with healthy donor CD19 CAR-T cells.^24^ In that study, we evaluated the therapeutic effect of CD8αβ killer iPS-T cells that were retrovirally modified by CAR and IL-15/IL-15Rα genes; however, the cells showed poor effectiveness in a subcutaneous tumor-bearing animal model. In the present study, we enhanced the anti-tumor activity of CD8αβ killer iPS-T cells against solid tumors by selecting the best combination of cytokine genes to be introduced into iPS-T cells.

Improvement of anti-tumor efficacy against solid tumors requires better migration, longer survival, and sustained cytotoxicity.^10^ These functions are regulated by the expression of genes related to chemotaxis, cell-cycle progression, and cytotoxicity; therefore, various types of gene editing have been attempted.^12^ Several studies have reported that cytokines enhance T cell functions through the phosphorylation of STATs, which are involved in the transcription of various genes. In particular, STAT1 and STAT3 phosphorylation is essential for the formation of memory T cells.^16^^,^^33^^,^^34^^,^^37^^,^^38^^,^^39^^,^^40^ Among the many cytokines, IL-15 and IL-21, which are common cytokine receptor γc family cytokines that phosphorylate several STATs, are useful for the formation of memory T cells and improving T cell anti-tumor efficacy.^27^^,^^41^^,^^42^ In this study, we found that iCAR-T cells expressing IL-15/IL-21 showed enhanced CXCR3-related migration to the tumor site and highly expressed CD45RO, CD62L, and CCR7, which relate to a younger memory phenotype (Figure 6C). In addition, we found that IL-2Rγ, which is a cytokine receptor of IL-15 and IL-21, was significantly upregulated on iCAR-T cells expressing IL-15/IL-21 but not on primary CD8 CAR-T cells *in vitro* (Figure 5A). This upregulation may increase STAT phosphorylation in iCAR-T cells, causing the upregulation of downstream transcripts including CXCR3 to increase the number of infiltrating cells and survival in tumors.

The preferential involvement of STAT1, rather than STAT3 or STAT5, in the regulation of CXCR3 is supported by ChIP-qPCR and our *in silico* analyses. Although IL-15 and IL-21 trigger multiple STAT pathways, a JASPAR analysis revealed that the proximal promoter of CXCR3 contains binding motifs with significantly higher affinity scores for STAT1 compared with other STAT members, suggesting that the CXCR3 promoter is intrinsically configured to preferentially respond to STAT1 activation (Figure S6C). This structural preference likely explains why STAT1 serves as the dominant transcription driver of tumor-homing machinery in iCAR-T cells expressing IL-15/IL-21.

An scRNA-seq analysis showed that iCAR-T cells expressing IL-15/IL-21 formed three clusters that were different from other iCAR-T cells. Those three clusters contain gene sets related to cell-cycle-, chemotaxis/Notch2-, and PD-1-related genes (Figure 7I). T cell exhaustion represents a significant challenge in CAR-T cell therapy for solid tumors.^4^^,^^43^ Although iCAR-T cells were successful in reducing exhaustion by expressing IL-15/IL-21, an analysis of gene expression revealed that the cells were ultimately exhausted (Figure 7H). The elimination of the gene clusters identified by scRNA-seq that cause T cell exhaustion is expected to enhance iCAR-T cell function. It is reported that notch signaling promotes CD8 memory T cell generation, which supports our hypothesis that tumor-infiltrating iCAR-T cells expressing IL-15/IL-21 change from a proliferating status to a chemotactic status to generate memory-like T cells by enhancing notch signaling.^44^^,^^45^^,^^46^ iCAR-T cells expressing IL-15/IL-21, but not those expressing only one of these cytokines, significantly enhanced the phosphorylation of STAT1, STAT3, and STAT5, which may explain the distinct clusters. The cellular profile found in the STAT signaling assay and gene expression profile of each cluster identified by the RNA velocity analysis will provide important insights in tumor immunology and will be considered in future studies.

In patients, the TME consists of not only tumors but also immune cells, stromal cells, and the extracellular matrix surrounding the tumor cells. The TME is known to affect the therapeutic efficacy of T cell immunotherapy. For example, solid tumors have been reported to suppress the function of T cells and other immune cells via TGF-β in the TME.^47^ At the same time, it has been reported that IL-12 and IL-18 can overcome the TME.^48^^,^^49^^,^^50^ However, the present study reveals that constitutive IL-12 expression significantly reduces the proliferative capacity of iCAR-T cells. In contrast, we previously reported that cytokines, including IL-12, effectively promote feeder-free expansion of iCAR-T cells.^25^ Therefore, subsequent studies should induce cytokine gene expression in iCAR-T cells upon activation, such as that achieved by synNotch, to enhance iCAR-T cell function.^51^

Notably, the divergence between short-term tumor control and long-term survival in the IL-15/IL-18 group highlights the importance of T cell fitness and persistence. While IL-15/IL-18 signaling provides sufficient initial activation to suppress tumor growth, our CyTOF data indicate a failure to establish a robust memory T cell pool within the TME (Figure S4). Furthermore, the relatively modest upregulation of CXCR3 and poor migratory response to CXCL9, CXCL10, and CXCL11 iCAR-T cells expressing IL-15/IL-18 limit the ability of these cells to continuously infiltrate and survey the tumor mass. In contrast, IL-15/IL-21 signaling synergistically programs iCAR-T cells toward a memory-like state with superior trafficking capabilities, ensuring sustained anti-tumor surveillance and significantly prolonged survival. These findings underscore the critical role of IL-15/IL-21 signaling in optimizing the functional fitness of iCAR-T cells, offering a promising strategy to overcome the current limitations of cell therapy in solid tumors.

Consistently, we showed that the combination of IL-15 and IL-21 in iCAR-T cells improves function, consistent with IL-15 and IL-21 enhancing T cell function and helping overcome the TME. In a syngeneic mouse model, T cells expressing murine IL-15 have been reported to promote the activation of natural killer (NK) cells within solid tumors and decrease the cell number of anti-inflammatory M2 macrophages.^52^ It has also been reported that murine IL-21 promotes the proliferation of CD8 T cells in solid tumors while decreasing the expression of PD-1 and TIM3, which are immune checkpoints, and also the number of regulatory T cells.^53^^,^^54^^,^^55^ We speculate that the secretion of IL-15 and IL-21 from iCAR-T cells maintains or improves the efficacy against solid tumors by positively affecting not only iCAR-T cells themselves but also surrounding immune cells in the TME. Furthermore, we propose that the STAT1-CXCR3 axis identified in this study plays a pivotal role in the TME. Synergistically enhanced STAT1 signaling likely boosts IFN-γ production by iCAR-T cells, which in turn can stimulate endogenous myeloid cells to secrete CXCR3 ligands (CXCL9, CXCL10, CXCL11). This effect would establish a self-amplifying recruitment loop, which is absent in the NSG model, whereby infiltrated iCAR-T cells facilitate the influx of both therapeutic and endogenous effector cells, potentially overcoming the cold tumor phenotype often encountered in clinical settings.

In addition, we found that iCAR-T cells expressing IL-15/IL-21 infiltrating solid tumors expressed many genes that contribute to T cell function. In particular, the expression of CCR5-related chemokine ligands, such as CCL3, CCL3L1, and CCL5, were upregulated on tumor-infiltrating iCAR-T cells expressing IL-15/IL-21, indicating that the number of migrating CD8 T cells, macrophages, and dendritic cells may increase in the presence of iCAR-T cells expressing IL-15/IL-21. An *in silico* analysis of transcription factor binding to the regulatory elements of CCL3, CCL3L1, and CCL5 genes in CD8 T cells predicted that STAT1 and STAT5B, which are significantly phosphorylated in iCAR-T cells expressing IL-15/IL-21, affect the expression of the three genes (Tables S2 and S3). This finding suggests that infiltrating iCAR-T cells expressing IL-15/IL-21 recruit a variety of immune cells to solid tumors and contribute to the formation of tertiary lymphoid structures, which benefit therapeutic efficacy against solid tumors.^56^^,^^57^

The advantages of using allogeneic immune cell products, such as PB-, cord blood-, or iPSC-derived CAR-T cells and CAR-NK cells, are under evaluation in several clinical trials.^58^^,^^59^ All these efforts aim to solve problems related to limited supply, unstable quality control, and the high cost of autologous immune cell therapy products while maintaining efficacy against tumors. Several groups including ours have applied genetic modification to produce immune cell products of higher proliferation capacity, but this leads to concern about uncontrolled growth (leukemogenesis) in patients. Additional genetic modification of the cellular products with suicide genes, such as tEGFR, iCas9, and HSV-TK, may solve this problem.^60^^,^^61^^,^^62^^,^^63^

Clinical CAR-T cell products typically consist of a mixed population of CD4^+^ and CD8^+^ T cells to leverage their synergistic effects. In contrast, current iPSC differentiation protocols predominantly yield cells of the CD8^+^ lineage, as the robust generation of mature CD4^+^ T cells from iPSCs remains a significant technical challenge in the field.^24^^,^^64^^,^^65^ In the present study, iCAR-T cells expressing IL-15/IL-21 showed robust anti-tumor efficacy against solid tumors. This finding underscores the critical role of IL-21, which is mainly expressed from CD4^+^ T cells, for iCAR-T cell function. Notably, our laboratory has recently succeeded in generating functional iPSC-derived CD4^+^ T cells.^66^ The findings emphasize the significance of IL-21, indicating that the established iPSC-derived CD4^+^ T cells have considerable potential. It is anticipated that these cells will function as a highly effective physiological partner in future iCAR-T cell therapy strategies.

Finally, our *in vivo* model used immunodeficient mice. These mice are deficient in T cells, NK cells, and myeloid-derived suppressor cells that are present in the human body. Therefore, a detailed evaluation of the remodeling of the TME through which iCAR-T cells might activate endogenous immunity remains to be conducted. To further validate the clinical translatability of our findings, experiments using syngeneic mouse models or humanized mouse models are necessary.

In summary, we investigated the effects of combinations of cytokine genes expressed in iCAR-T cells and found that IL-15 and IL-21 synergistically improved iCAR-T cell function and revealed that the STAT1-CXCR3 axis can modulate the TME. Although IL-15 and IL-21 activate multiple STAT proteins, their combined signaling preferentially drives the phosphorylation of STAT1. Activated STAT1 binds to the CXCR3 promoter region, thus functioning as a molecular switch for tumor homing. By sustaining high CXCR3 expression, IL-15/IL-21-engineered iCAR-T cells can effectively sense and migrate along CXCL9, CXCL10, and CXCL11 gradients commonly present in solid tumors, overcoming a major barrier to CAR-T therapy. Accordingly, tumor-infiltrating iCAR-T cells expressing IL-15/IL-21 proliferated, survived with the memory phenotype, and showed enhanced anti-tumor efficacy against solid tumors. In total, this study is expected to contribute to the development of cancer immunotherapies against solid tumors using iCAR-T cells, which are uniform in quality and suitable for mass production as a platform of allogeneic cell therapeutic products.

## Materials and methods

### Cell lines

Huh7 cells (RRID: CVCL_0336) were purchased from RIKEN BRC (Japan). Huh7, JHH7 (RRID:CVCL_2805), and SK-Hep-1 cells (RRID: CVCL_0525) transduced with mock and GPC3 cells were maintained in Dulbecco’s modified Eagle’s medium supplemented with 10% fetal bovine serum (FBS) and Penicillin-Streptomycin-Glutamine (PSG) solution (Sigma, USA). The cells were cultured at 37°C, 5% CO_2_.

The T cell-derived iPS cell clone (TkT3V1-7) was established from T cells of a healthy donor, cultured on Easy iMatrix-511 silk (Nacalai Tesque, Japan)-coated plates, and maintained in StemFit AK02N medium (Ajinomoto, Japan). The cells were cultured at 37°C, 5% CO_2_. All cell lines were routinely screened for mycoplasma contamination, and only negative cultures were used for experiments.

### Generation of GPC3-CAR-expressing iPSCs

TkT3V1-7 cells were transduced in 24-well plates with a lentivirus vector (CS-UbC-GC33-T2A-tEGFR) as described previously.^21^ tEGFR^+^ TKT3v1-7 cells were evaluated as GPC3-CAR-expressing cells and sorted and expanded by fluorescence-activated cell sorting (FACS).

#### Generation of STAT1-KO iPSCs and CXCR3-KO iPS-T cells

We made the STAT1-KO vector (Figure 5F) using 4D-Nucleofector (Lonza, Switzerland), aP4 Primary Cell 4D-Nucleofector X Kit S (Lonza), and three different guide RNAs (gRNA1: 5′-AGACAGCAGAGCGCCTGTATTGG-3′, gRNA2: 5′-TTAATGATGAACTAGTGGAGTGG-3′, and gRNA3: 5′-CAGCTGATCCAAGCAAGCATTGG-3′). The same was done for the CXCR3-KO but the three gRNAs were gRNA1: 5′-CCAAGTGCTAAATGACGCCG-3′; gRNA2, 5′-GGAAGGCCCGGTCGAAGTTC-3′, and gRNA3, 5′-GGGGCCCCCGGCGGTAGAGC-3′) and electroporate by MaxCyte (USA). gRNAs were synthesized using a CUGA7 gRNA Synthesis Kit (Nippon Gene, Japan), and TrueCut Cas9 Protein version 2 (Thermo Fisher, USA) was transfected according to the manufacturer’s instructions.

### Differentiation of CAR iPSCs

#### iPSCs to HSCs

To initiate differentiation, a total of 3.0 × 10^5^ iPSCs were used. We suspended the iPSCs in StemFit AK02N medium (Ajinomoto) supplemented with 10 μM Y-27632 (FujiFilm Wako, Japan) and 10 μM CHIR99021 (Tocris Bioscience, UK) and then cultured them in 6-well ultra-low attachment plates (Corning, USA) for 24 h (day 0). After 24 h, the cells were collected and cultured in EB medium: StemPro-34 (Thermo Fisher Scientific, US) supplemented with 2 mM PSG solution (Sigma), 2 mM GlutaMAX (Thermo Fisher Scientific), 50 μg/mL ascorbic acid-2-phosphate (Sigma), 400 μg/mL monothioglycerol (Nacalai Tesque), and 1× insulin-transferrin-selenium (ITS) solution (Thermo Fisher Scientific). Then, 50 ng/mL rhBMP-4 (Miltenyi Biotec, Germany), 50 ng/mL rhVEGF (vascular endothelial growth factor; FujiFilm Wako), and 50 ng/mL rh basic fibroblast growth factor (rhbFGF) (FujiFilm Wako) were added to each well (day 1). After 24 h, 6 μM SB431542 (FujiFilm Wako) was added (day 2). After 48 h, the differentiating EBs were collected, washed with PBS (Nacalai Tesque), and cultured with 2 mL EB basal medium supplemented with 50 ng/mL rhVEGF, 50 ng/mL rhbFGF, and 50 ng/mL rh stem cell factor (SCF) (FujiFilm Wako) per well (day 4). After 48 h, the differentiating EBs were collected, washed with PBS, and cultured with 2 mL EB basal medium supplemented with 50 ng/mL rhVEGF, 50 ng/mL rhbFGF, 50 ng/mL rhSCF, 30 ng/mL rh thrombopoietin (PeproTech, USA), and 10 ng/mL FLT3L (FujiFilm Wako) per well (day 6). EB basal medium with the supplements was refreshed on days 8 and 10. The cells were cultured in 5% CO_2_/5% O_2_/90% N_2_ until day 6, and then in 5% CO_2_ until day 11.

### Differentiation

#### HSCs to iCAR-T cells

Day 11-differentiated HSCs were co-cultured with delta-like 1-expressing OP9 feeder cells. The cells were then co-cultured in α-minimum essential medium (MEM) (Sigma) supplemented with 15% FBS, 2 mM PSG, 1× ITS solution, 50 ng/mL ascorbic acid 2-phosphatase, 10 ng/mL hFlt3L, and 1 ng/mL rhIL-7 (PeproTech). After 21 days of co-culture, iPS-T cells were stimulated with 500 ng/mL OKT3 (eBioscience, USA). The cells were maintained in α-MEM supplemented with 15% FBS, 1× ITS-G, 2 mM PSG, 50 ng/mL ascorbic acid 2-phosphatase, and 10 ng/mL rhIL-7. After 2 days of stimulation with OKT3, the cells were harvested on retronectin (Takara, Japan)-coated 48-well plates. The cells were then cultured in α-MEM supplemented with 15% FBS, 2 mM PSG, 50 ng/mL ascorbic acid 2-phosphatase, 1× ITS-G, 10 ng/mL rhIL-7, and 10 ng/mL rhIL-21 (PeproTech). After 5 days of culture, CD5^+^CD8b^+^ iCAR-T cells were sorted by FACS and expanded using 2 μg/mL phytohemagglutinin (PHA) stimulation. The cells were cultured in α-MEM supplemented with 15% FBS, 2 mM PSG, 50 ng/mL ascorbic acid 2-phosphatase, 1× ITS-G, 5 ng/mL rhIL-7, and 5 ng/mL rhIL-15 (PeproTech) and cultured at 37°C, 5% CO_2_.

#### Flow cytometry analysis and antibodies

We used an LSR (BD Biosciences, USA) or FACS AriaII Flow Cytometer (BD Biosciences). The data were analyzed using FlowJo software (Tree Star, USA; RRID: SCR_008520). For the analysis, singlet cells were separated, and live cells were detected by propidium iodide-negative (PI^−^) cells. The following antibodies were used for the staining and analysis of the cells: AF700-CD3 (clone UCHT1, BD Biosciences; RRID: AB_396952); BV510-CD4 (clone OKT4, BioLegend, USA; RRID: AB_2561377); PE-Cy7-CD5 (clone UCTH2, eBioscience; RRID: AB_1582282); PerCPCy5.5-CD8 (clone SK1, BioLegend; RRID: AB_1967149); APC-CD8β (clone 2ST8.5H7, BD Biosciences; RRID: AB_1645747); FITC-CD14 (clone M5E2, BioLegend; RRID: AB_314188); PE-Cy7-CD34 (clone 4H11, Abcam, UK); APC-CD43 (clone 1G10, BD Biosciences; RRID: AB_1645460); BV510, FITC, and APC-Cy7-CD45 (clone HI30, BioLegend; RRIDs: AB_2561940, AB_314394, and AB_314402); APC-CD45RA (clone 5H9, BD Biosciences; RRID: AB_398468); BV510-CD45RA (clone HI100, BioLegend; RRID: AB_2561947); BV510 and APC-Cy7-CD45RO (clone UCHL1, BioLegend; RRIDs: AB_2565801 and AB_10895897); PE-Cy7-CD62L (clone DREG-56, BioLegend; RRID:AB_830801); BV421-CD122 (IL-2Rβ) (clone TU27, BioLegend; RRID: AB_2561835); APC-CD132 (IL-2Rγ) (clone TUGh4, BioLegend; RRID: AB_2123584); APC-CD215 (IL-15Rα) (clone JM7A4, BioLegend; RRID: AB_2561440); Pacific Blue-CD235a (clone HI264, BioLegend; RRID: AB_11218988); PE-Cy7-CD360 (IL-21Rα) (clone 17A12, BioLegend; RRID: AB_2721662); PerCPCy5.5 and APC-CCR7 (clone G043H7, BioLegend; RRIDs: AB_10916121 and AB_10917387); AF647-CCR5 (clone HEK/1/85a, BioLegend; RRID: AB_528760); Pacific Blue and PE-Cy7-CXCR3 (clone G025H7, BioLegend; RRIDs: AB_2561442 and AB_11219383); BV421-PD-1 (clone EH12.2H7, BioLegend; RRID: AB_10960742); APC-LAG-3 (clone 7H2C65, BioLegend; RRID: AB_2728373); PE-Cy7-TIM3 (clone F38-2E2, BioLegend; RRID: AB_2561720); PE and APC EGFR (clone AY13, BioLegend; RRIDs: AB_10896794 and AB_11150410); and APC-αβTCR (clone IP26, BioLegend; RRID: AB_10612569). For staining with annexin V, we used an Annexin V-APC Assay Kit (Abcam, catalog no. ab236215). To inhibit STAT1 phosphorylation, we used 100 nM pravastatin (Cayman, USA) or 100 nM fludarabine (Cayman).

#### Cell-cycle progression and cell counts

We used Cell Cycle Assay Solution Deep Red (Dojindo, Japan) according to the manufacturer’s instructions to analyze the cell cycle. A total of 5.0 × 10^5^ iCAR-T cells were cultured in α-MEM supplemented with 15% FBS, 2 mM PSG, 1× ITS-G, and 50 ng/mL ascorbic acid 2-phosphatase in the presence of JHH7 cells at an E:T ratio of 5:1. We added 10 ng/mL rhIL-12 (PeproTech), rhIL-15, rhIL-18 (PeproTech), or rhIL-21 to the culture medium. The iCAR-T cell number was measured using Precision Count Beads (BioLegend) according to the manufacturer’s instructions.

#### Generation of cytokine-expressing iCAR-T cells

Here, we discuss the construction of retrovirus vector containing cytokine-encoding gene or shRNA. To generate cytokine-expressing iCAR-T cells, we genetically inserted cytokine-encoding genes in a pMY retrovirus vector (Cell Biolabs, USA; RRID: Addgene_163351). IL-12A, IL-12B, IL-15, and IL-18 mRNA were obtained from PB mononuclear cells (PBMCs) stimulated with LPS. IL-21 mRNA was obtained from PBMCs stimulated with PHA. We reverse transcribed mRNA using a PrimeScriptII 1^st^ Strand cDNA Synthesis Kit (Takara). We amplified the cytokine-encoding genes using KOD Plus *NEO* enzyme (Toyobo, Japan). The IL-15 and IL-18 signal sequences were replaced with the IL-2 signal sequence. Each cytokine and mCherry gene was linked by an internal ribosome entry site sequence. The constructed genes were genetically inserted into the pMY retrovirus vector using an In-Fusion HD Cloning Kit (Takara). For shRNA vectors, we inserted the following shRNA gene sequences into pMY vectors using an In-Fusion HD Cloning Kit (Takara): STAT3-shRNA1, 5′-GGCGTCCAGTTCACTACTAAA-3′; STAT3-shRNA2, 5′-CTCAGAGGATCCCGGAAATTT-3′; STAT5-shRNA1, 5′-GTACTACACTCCTGTGTG-3′; and STAT5-shRNA2: 5′-GCAACAGGCCCATGACC-3′.

#### Generation of retroviruses

GP293 cells were harvested on a 10-cm dish 2 days before transfection. Two days later, we transfected the cytokine gene-encoded retrovirus vector and vesicular stomatitis virus G protein gene-encoded pMD2.G vector (Addgene, USA; RRID: Addgene_12259,) using DNA transfection reagents. We generated the recombinant retroviral vector as previously reported.^24^ For the transduction, we stimulated iCAR-T cells with 2 μg/mL PHA. After 3 or 4 days, the activated iCAR-T cells were harvested on retrovirus supernatant in a 50-μg/mL retronectin (Takara)-coated 48-well plate. iCAR-T cells were treated according to the manufacturer’s instructions.

#### Cytokine production

An IL-12 p70 Human Uncoated ELISA Kit with Plates (Invitrogen, USA), an IL-15 Human Uncoated ELISA Kit with Plates (Invitrogen), and an IL-21 Human Uncoated ELISA Kit with Plates (Invitrogen) were used to measure IL-12, IL-15, and IL-21, respectively. A Human IL-18 ELISA Kit (Abcam) was used to measure IL-18. A total of 5 × 10 iCAR-T cells were maintained in α-MEM supplemented with 15% FBS, 1× ITS-G, 2 mM PSG, 1 ng/mL rhIL-7, and 50 ng/mL ascorbic acid 2-phosphatase. Cell culture supernatants were collected after 48 h, centrifuged, and frozen until the time of the assay.

To measure human IL-2, IFN-γ, and TNF released from iCAR-T cells, a BD Cytometric Bead Array Flex Set System (BD Biosciences) was used. A total of 1.0 × 10^6^ resting iCAR-T cells were maintained in α-MEM supplemented with 15% FBS, 2 mM PSG, 1× ITS-G, and 50 ng/mL ascorbic acid 2-phosphatase and cultured in the presence of skHep-1-vec and skHep-1-GPC3 cells at a 1:1 ratio. Cell culture supernatants were collected at 72 h, centrifuged, and frozen until the time of the assay.

#### Cytotoxicity assay

We used the Non-Radioactive Cellular Cytotoxicity Assay Kit (Techno-Suzuta, Japan) according to the manufacturer’s instructions. Huh7, JHH7, skHep-1-vec, and skHep-1-GPC3 cells were pulsed with Bis-acetoxymethyl Hydroxy-Terephthalate (BM-HT) reagent at 37°C for 15 min and washed three times, and 5 × 10^5^ cells were seeded into a 96-well plate. Effector cells were loaded into the wells at E:T ratios ranging from 1.25:1 to 10:1 and cito-cultured for 3 h. After the co-culture, we mixed the culture medium and centrifuged. Then, 20 μL co-culture supernatant was mixed with 200 μL Eu solution (PerkinElmer, USA), and time-resolved fluorescence was measured through an NIVO plate reader (PerkinElmer). In Figure S3B, luciferase expressing skHep-1-GPC3 and skHep-1-vec cells were used. Effector cells were loaded into the wells at E:T ratios ranging from 2.5:1 to 20:1 and co-cultured for 24 h. To evaluate cytotoxicity, luminescence was evaluated by an NIVO plate reader (PerkinElmer). The cells were cultured on α-MEM supplemented with 15% FBS and 2 mM PSG at 37°C, 5% CO_2_.

To evaluate the change of cytotoxic activity over time, we used an xCelligence RTCA HT Analyzer (Agilent Technologies, USA). We cultured 5 × 10^3^ skHep-1-GPC3 cells on a 96-well E-Plate. After 24 h, we co-cultured the cells with 1.5 × 10^4^ cytokine-expressing iCAR-T cells on α-MEM supplemented with 15% FBS, 2 mM PSG, and 1 ng/mL rhIL-7 at 37°C, 5% CO_2_.

#### Cell chemotaxis

The chemotaxis of iCAR-T cells was assayed in 5-μM pore size transwells inserted with polycarbonate membranes (Corning, USA). Control (α-MEM and 1% BSA) or medium containing 200 nM hCXCL9 (PeproTech), hCXCL10 (PeproTech), and hCXCL11 (PeproTech) was placed at the bottom of the 96-wells. A total of 5.0 × 10^5^ iCAR-T cells were placed on the upper wells and incubated at 37°C for 3 h. Supernatants of JHH7 and skHep-1-GPC3 were made from 1 × 10^6^ cells harvested in 10-cm dishes and cultured under α-MEM conditions for 2 days. Monoclonal mouse IgG1 against human CXCR3 (clone 49801, R&D Systems, USA, catalog no. MAB160,) was used to evaluate CXCR3-dependent migration. A TC20 automated cell counter (Bio-Rad, USA) was used to count iCAR-T cells.

#### Western blotting

Lysate samples were obtained from cytokine-expressing iCAR-T cells treated with RIPA buffer (FujiFilm Wako Chemicals) containing Halt Protease and phosphatase inhibitor single-use cocktail (100×) (Thermo Fisher Scientific). For western blotting, we used Wes (Protein Simple, USA). Glyceraldehyde 3-phosphate dehydrogenase (GAPDH) and STAT1, STAT3, and STAT5 protein expression were observed using a 12- to 230-kDa Jess or Wes Separation Module 8 × 25 capillary CAR-Tridges (Protein Simple) according to the manufacturer’s instructions. To observe the proteins, the following antibodies were used: GAPDH rabbit monoclonal antibody (mAb) (clone: 14C10, RRID: AB_561053, Cell Signaling Technology, USA), p-STAT1 (Tyr701) rabbit mAb (clone: 58D6, RRID: AB_561286, Cell Signaling Technology), p-STAT3 (Tyr705) rabbit mAb (clone: D3A7, RRID: AB_10140544, Cell Signaling Technology), p-STAT5 (Tyr694) rabbit mAb (clone: C11C5, RRID: AB_10200754, Cell Signaling Technology), STAT1 rabbit mAb (clone: D1K9Y, RRID: AB_2737027, Cell Signaling Technology), STAT3 rabbit mAb (clone: D3Z2G, RRID: AB_2629499, Cell Signaling Technology), and STAT5 rabbit mAb (clone: D2O6Y, RRID: AB_2737403, Cell Signaling Technology). Compass software (Protein Simple) was used to evaluate the relative expression of STAT1, STAT3, and STAT5.

STAT1-KO iPSC samples were treated with radioimmunoprecipitation assay (RIPA) buffer (FujiFilm Wako Chemicals). Samples were evaluated using 12- to 230-kDa Jess or Wes Separation Module 8 × 25 capillary CAR-Tridges (Protein Simple), GAPDH rabbit mAb (clone: 14C10, Cell Signaling Technology), and STAT1 rabbit mAb (clone: D1K9Y, Cell Signaling Technology) according to the manufacturer’s instructions.

#### ChIP-qPCR assay

A SimpleChIP Enzymatic Chromatin IP Kit (Magnetic Beads) (Cell Signaling Technology), anti-STAT1, anti-STAT3, and anti-STAT5 antibodies, and normal rabbit IgG no. 2729 (control; RRID: AB_2617119, Cell Signaling Technology) were used to observe STAT1, STAT3, and STAT5 binding to the CXCR3 promoter region. A total of 1.2 × 10^6^ iCAR-T cells and primary CD8 CAR-T cells were used in this study. After preparing the samples, qPCR was performed using designed primers and PowerUp SYBR Green Master Mix (Thermo Fisher) using the standard cycling mode. The ChIP-qPCR primers used were region 1: forward, 5′-CAGACAGCCGTGGGATCAAT-3′, reverse, 5′-AGGTGCTTTCTCCGACACTG-3′; region 2: forward, 5′-AAGCTGGGCCTGATTCTGTC-3′, reverse, 5′-AAGTCTGTGGTGGGCTTCTG-3′; and region 3: forward, 5′-AAGCAGGTAAAGTCCAGCCC-3′, reverse, 5′-TGCCAAGTAACGTTCCTCCC-3′.

#### ATAC-seq analysis

ATAC-seq was performed by Rhelixa (https://www.rhelixa.com/). Chromatin accessibility peaks near the CXCR3 genome region are shown in Integrative Genomics Viewer (https://igv.org/doc/desktop/).

### *In vivo* animal models

#### Bioluminescence imaging

Female NSG mice, 6–10 weeks old (RRID: MGI:3770662), purchased from Oriental Bio (Japan) were used in the experiments. For the imaging, 2.5 × 10^6^ skHEP-1-GPC3 cells or 1.0 × 10^6^ JHH7 cells were subcutaneously injected in the left flanks of the mice on day −10 or day −14, respectively. Animals were assigned to experimental groups using simple randomization. iCAR-T cells were intravenously injected on day 0.

The mice were imaged for tumor bioluminescence regularly by the intraperitoneal injection of luciferin. Bioluminescence imaging was performed using an IVIS Lumina III (PerkinElmer).

#### scRNA-seq of tumor-infiltrating T cells

Eight-week-old female NSG mice purchased from Oriental Bio were used for the experiments. We subcutaneously injected 1.0 × 10^6^ JHH7 cells into the left flanks of the mice on day −14. Animals were assigned to experimental groups using simple randomization. iCAR-T and primary CD8 CAR-T cells were intravenously injected on day 0. Tumor-infiltrating cells were sorted by hCD45 and mCherry expression on day 14. After sorting, the cells were treated with a Chromium Single-Cell 5′ Library and Gel Bead Kit (10x Genomics) according to the manufacturer’s protocol. Each sample from the scRNA-seq library was sequenced using a NovaSeq 6000 (Illumina) with a NovaSeq 6000 S2 flow cell (Illumina). To make FASTQ files, we used bcl2fastq (version 2.20.0.422) and Cell Ranger (version 6.1.2) as the default setting. Sequence data results are presented in Seurat format derived from Loupe Browser 6 (10x Genomics) using refdata-gex-GRCh38-2020-A(10x Genomics) as reference. Using significantly expressed genes on each cluster, pathway analysis was done on Reactome (https://reactome.org/PathwayBrowser/#/).

#### Transcription factor analysis

An *in silico* analysis of the transcription factors that regulate CXCR3, CCL3, CCL3L1, and CCL5 expression were performed on ChIP-Atlas Enrichment Analysis (https://chip-atlas.org/) (RRID: SCR_015511) and JASPAR (https://jaspar.genereg.net/analysis) (RRID: SCR_003030). The CCL3, CCL3L1, and CCL5 promoter regions upstream of the transcription starting sites have been reported elsewhere.^67^^,^^68^

#### Analysis of tumor-infiltrating iCAR-T cells

NSG mice were sacrificed on days 2, 10, and 14 to evaluate the migration and memory phenotype of tumor-infiltrating T cells. One tumor fraction was digested gently with an MACS dissociator and Precellys Evolution (Bertin Instruments, France). After the digestion, the fraction was filtered through a 70-mm nylon mesh cell strainer, and red blood cells were lysed and analyzed with flow cytometry. Other fractions were prepared for histopathological analysis. For mass spectrometry analysis, we used the Maxpar Complete Human T Cell Immuno-oncology Panel Set to analyze tumor-infiltrating T cells (Standard Biotools, USA). In this analysis, we added anti-mouse CD45 (30-F11)-89Y, Cell-ID Cisplatin-^198^Pt, and purified anti-human CD62L antibody (clone: DREG-56, BioLegend) labeled with ^145^Nd. Anti-CD62L labeling was done using a Maxpar X8 Antibody Labeling Kit, ^145^Nd according to the manufacturer’s instructions. After bead removal and singlet cell detection, live cells were evaluated using Cisplatin-^198^Pt negative cells. For CyTOF analysis, we performed FlowSOM (RRID: SCR_016899) and t-distributed stochastic neighbor embedding (t-SNE) analysis using the FlowJo plug-in listed on https://www.flowjo.com/exchange/#/ and FlowJo version 10.10.0. In this study, we analyzed the expression of 34 proteins by CyTOF mass cytometry. The total dataset consisted of approximately 10,000 cells, requiring dimensionality reduction to explore the relationships among cell populations in high-dimensional space. For this purpose, we employed t-SNE, which is particularly effective in preserving local structures and accurately distinguishing cellular subsets. While UMAP offers faster computation, t-SNE was deemed more suitable for this analysis due to the moderate dataset size and the need for precise separation of cellular populations.

#### Therapeutic efficacy

A total of 5 × 10^5^ JHH7 cells or 2.5 × 10^6^ skHep-1-GPC3 cells were injected subcutaneously on days 3 and 10, respectively. We intravenously injected 5 × 10^6^ iCAR-T cells on days 0 and 7. Tumor dimensions were measured with calipers, and tumor volumes were calculated using the formula V = LW^2^/2, where L is length (longest dimension) and W is width (shortest dimension). Group sizes were determined based on pilot data and previous reports, and no formal statistical power calculation was performed. Animals were randomized. Investigators were not blinded during the injections, but tumor measurements were performed by an independent researcher to minimize bias.

### Statistical analyses

The figure captions state the number of independent experiments conducted, and data are represented as the mean ± standard deviation or the mean ± standard error of the mean (SEM) as indicated. All statistical analyses were performed using GraphPad Prism 8 software (RRID: SCR_002798). *p* values were calculated using one-way ANOVA with Tukey’s multi-comparison test or Student’s *t* test. Statistical significance was considered at ∗*p* < 0.05, ∗∗*p* < 0.01, ∗∗∗*p* < 0.005, and ∗∗∗∗*p* < 0.001.

## Data and code availability

scRNA-seq data were deposited in the NCBI_GEO repository (https://www.ncbi.nlm.nih.gov/geo/query/acc.cgi?acc=GSE252998) under accession number GSE252998 (BioProject PRJNA1055728, RRID: SCR_004801. No custom code was used in this study. ATAC-seq data were deposited to the NCBI_GEO repository (https://www.ncbi.nlm.nih.gov/geo/query/acc.cgi?acc=GSE306476) under accession number GSE306476.

## Acknowledgments

We thank the Kyoto University Center for anatomical, pathological, and forensic medical research and the CiRA Foundation and Rhelixa for their help with the immunohistochemical staining, scRNA-seq analysis, and ATAC-seq analysis. We also thank Mr. Akito Tanaka, Ms. Katsura Noda, Ms. Kanae Mitsunaga, Dr. Atsutaka Minagawa, and Dr. Tatsuki Ueda (Kyoto University) for their technical and administrative assistance and Drs. Yasushi Uemura, Tetsuya Nakatsura (National Cancer Center), and Prof. Koji Tamada (Yamaguchi University) for providing critical materials. This research was supported by 10.13039/100009619AMED under grant nos. JP22bm0104001, JP22ck0106741, and JP22bm1004001 and by a research grant from Shinobi Therapeutics. The entire study was conducted in accordance with the Declaration of Helsinki and permitted by the institutional ethical board of Kyoto University.

## Author contributions

A.I. designed, analyzed, and performed all experiments; contributed to the study design; and drafted the manuscript. M.W. and Y.K. discussed and guided this study. T.I. performed and helped with the animal experiments. S.K. designed, directed and supervised this research.

## Declaration of interests

S.K. is a founder, shareholder, and scientific adviser of Shinobi Therapeutics and received research funding from Takeda Pharmaceutical, Astellas, Panasonic Holdings, and Shinobi Therapeutics.

## Supplemental information

Document S1. Figures S1–S7 and Tables S1–S3

Document S2. Article plus supplemental information
