# Supplementary material for: IL-15 and IL-21 synergy improves anti-tumor efficacy of iPSC-derived cytotoxic T cells in solid tumors
Source: Mol Ther. 2026 May 12;34(8):4758–77. doi: 10.1016/j.ymthe.2026.04.036 (PMC13464151; doi:10.1016/j.ymthe.2026.04.036)
Supplement: Supplementary file 1 [file mmc1.pdf]

YMTHE, Volume *34*

## Supplemental Information

# IL-15 and IL-21 synergy improves anti-tumor efficacy of iPSC-derived cytotoxic T cells in solid tumors

**Akihiro Ishikawa, Masazumi Waseda, Tomoko Ishii, Yohei Kawai, and Shin Kaneko**

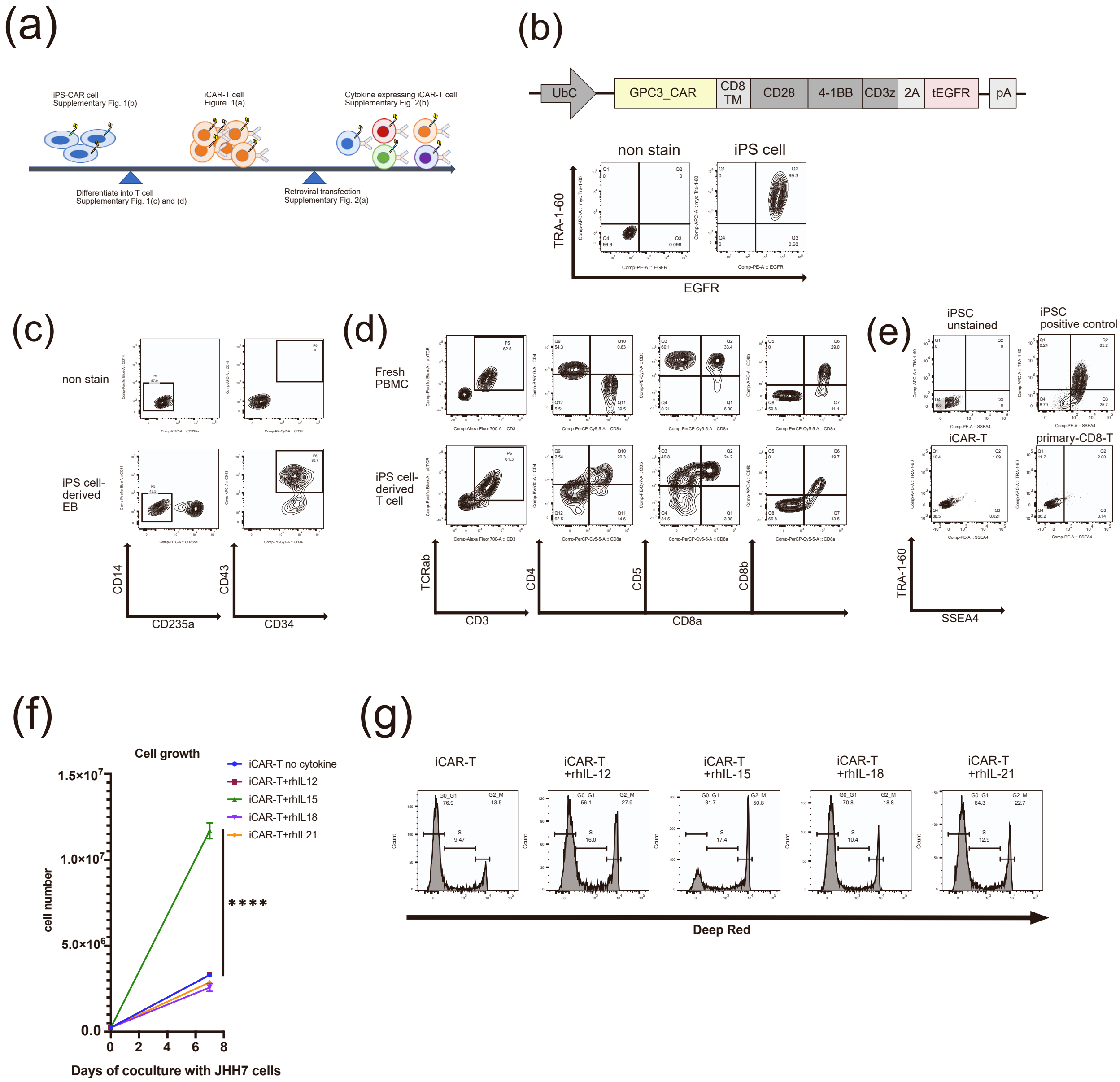


**Figure S1 rhIL-15 improved iCAR-T cell growth and cell cycle progression.** (a) Scheme of the T cell induction from a T-iPSC clone (TkT3V1-7). (b) Scheme of the lentiviral constructs encoding GPC3-CAR. GPC3-CAR was expressed with tEGFR gene and linked with the T2A sequence. Flow cytometry plots of hematopoietic (c) and (d) CD4+CD8+ double positive stages induced from TkT3V1-7. EB, embryoid bodies. (e) TRA-1-60 and SSEA4 expression on iCAR-T and primary CD8 T cells. (f) Representative growth and (g) cell cycle progression of iCAR-T cells co-cultured with JHH7 cells (n=3). 10 ng/mL rhIL-12, -15, -18, or -21 were added to the medium. ****P < 0.001; one-way ANOVA and Tukey's multiple comparison test.

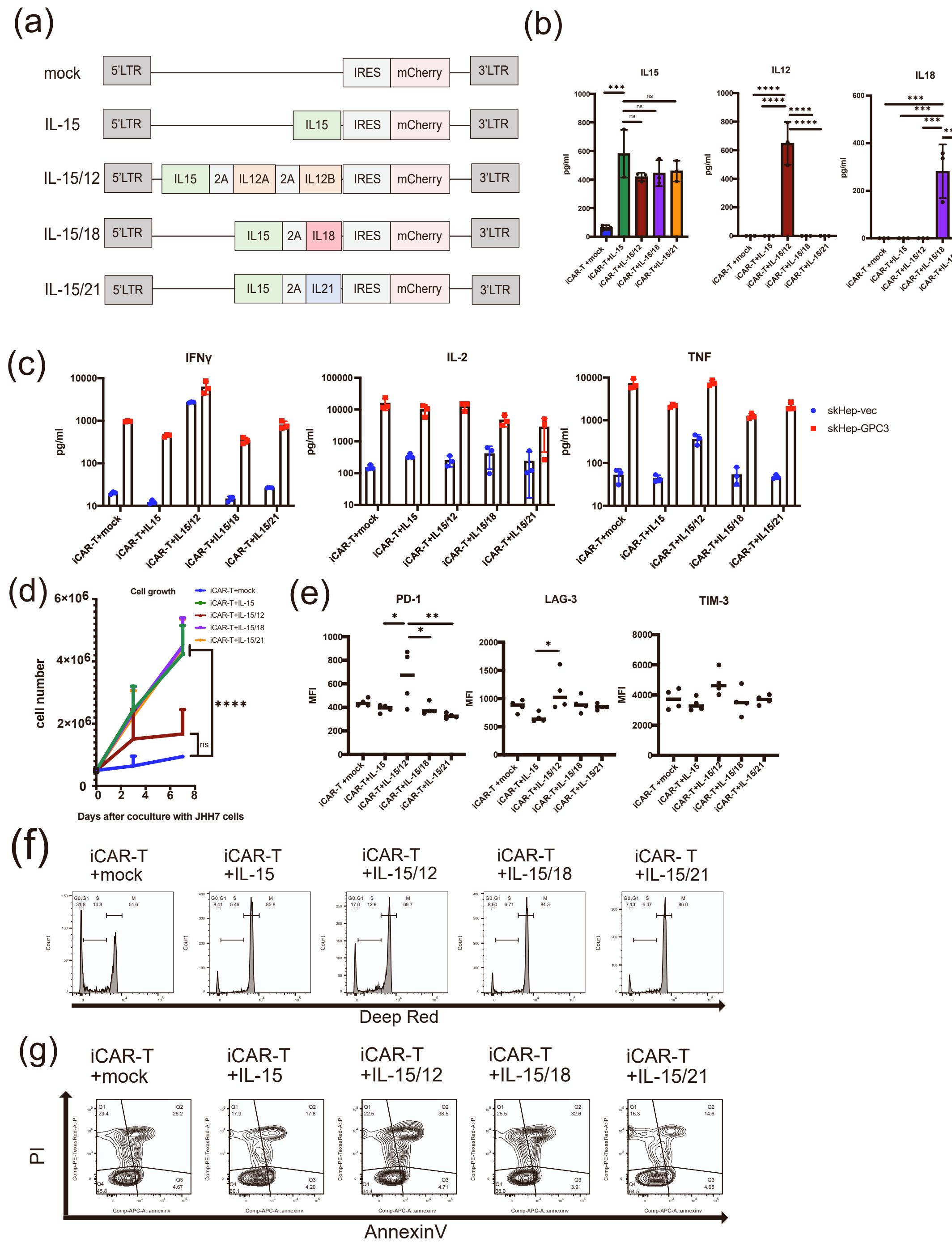

**Figure S2 Proliferation and anti-apoptotic potential of each cytokine-expressing iCAR-T cell types**

(a) Schematic of retroviral constructs encoding IL-15, -12, -18, and -21. The constructs also expressed mCherry gene, and the genes were linked with the IRES sequence. (b) Cytokine expression measured using ELISA. (c) Representative IFN-g, IL-2, and TNF expression in iCAR-T cells co-cultured with skHep-1-vec or skHep-1-GPC3 cells. (d) Representative growth ability of iCAR-T cells co-cultured with JHH7 cells (n=3). (e) Expression of immune checkpoint markers in iCAR-T cells after 7 days of co-culture with skHep-1-GPC3 cells (n=4). (f) Representative cell cycle progression of iCAR-T cells (n=3). (g) Representative flow cytometry plots of AnnexinV and PI positive cells (n=3). Data represent the mean ± SEM of n independent experiments. *P < 0.05, **P < 0.01, ***P < 0.005, ****P < 0.001; one-way ANOVA with Tukey's multiple comparison test.

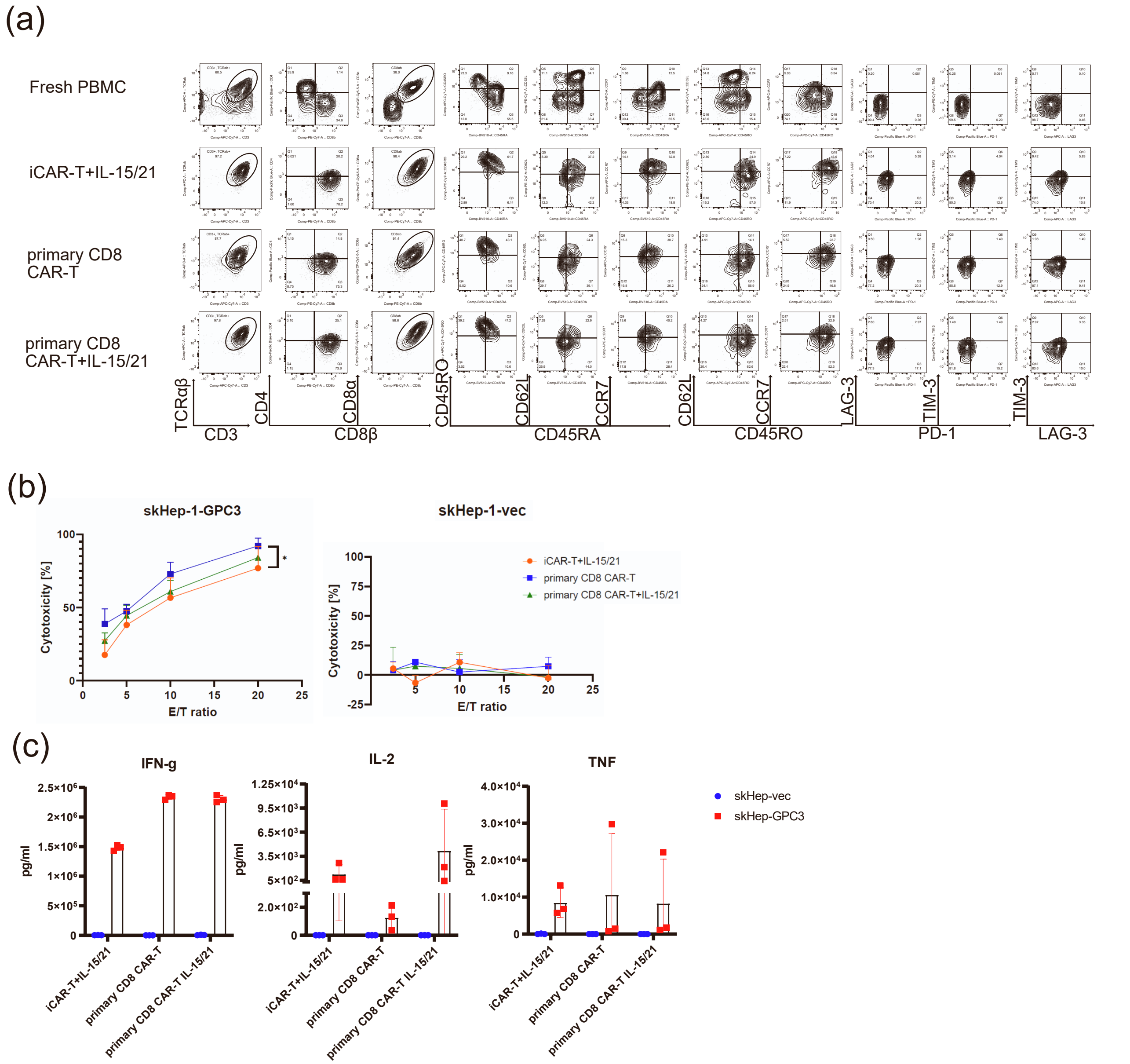


**Figure S3 Characterization of iCAR-T cells expressing IL-15/21 and primary CD8 CAR-T cells.**
(a) Representative flow cytometry plots of iCAR-T and primary CD8 CAR-T cells cell surface markers. (b) Cytotoxicity of iCAR-T cells against skHep-1-GPC3, and skHep-1-vec cells. (c) Representative IFN-g, IL-2, and TNF expression in iCAR-T cells expressing IL-15/21 and primary CD8 CAR-T cells co-cultured with skHep-1-vec or skHep-1-GPC3 cells.

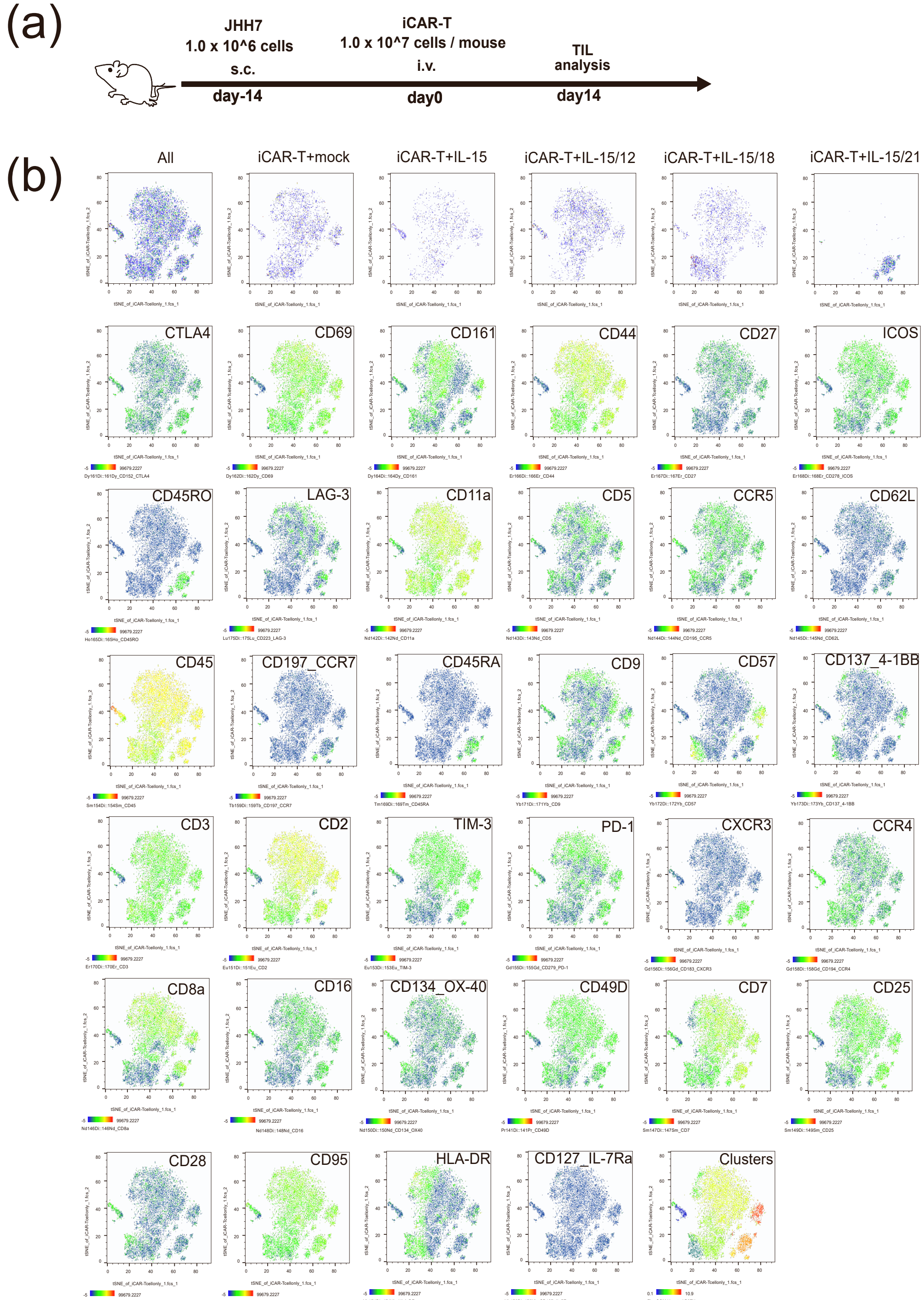


**Figure S4 Tumor-infiltrating iCAR-T cells expressing IL-15/21 have unique cell surface markers compared to other iCAR-T cells.**

(a) Schedule of the JHH7 cell inoculation and iCAR-T cell injection. 1.0 x 106 JHH7 cells were subcutaneously injected into the left back side of the mice. (b) tSNE plots of tumor-infiltrating iCAR-T cells cell surface markers.

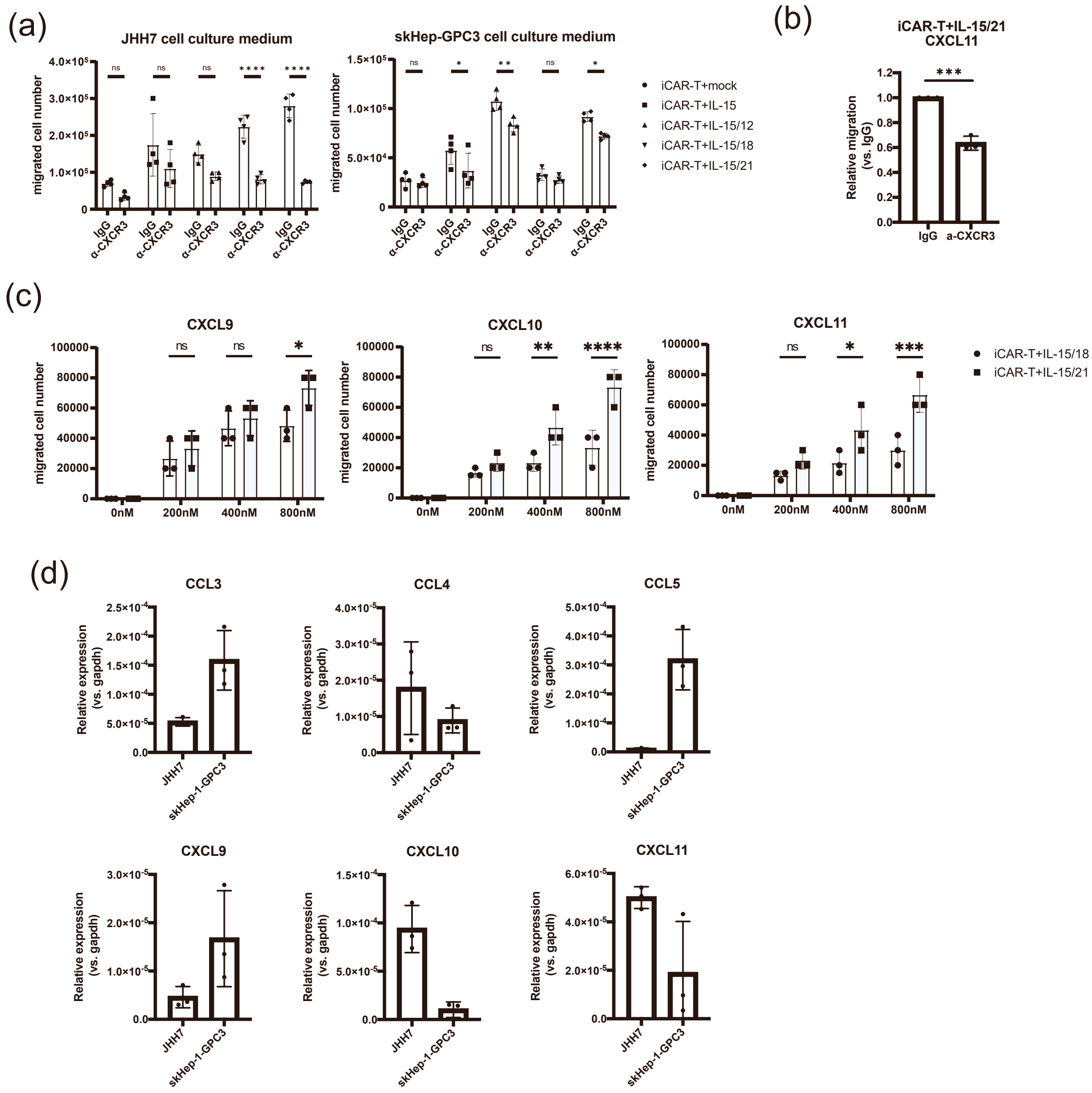


**Figure S5 CXCR3-related migration of iCAR-T cells expressing IL-15/21 under anti-CXCR3 antibody.**

(a) Migration activity of iCAR-T cells against JHH7 and skHep-1-GPC3 cell culture medium after treatment with IgG and anti-CXCR3 antibody. (b) Migration of iCAR-T cells expressing IL-15/21 against CXCL11 after treatment with IgG or anti-CXCR3 antibody. (c) Migration activity against different CXCL9, 10, and 11 concentrations of iCAR-T cells expressing IL-15/18 and IL-15/21. (d)Representative CCL3, CCL4, CCL5, CXCL9, CXCL10 and CXCL11 expression levels are shown. *P < 0.05, **P < 0.01, ***P < 0.005, ****P < 0.001; one-way ANOVA with Tukey's multiple comparison test.

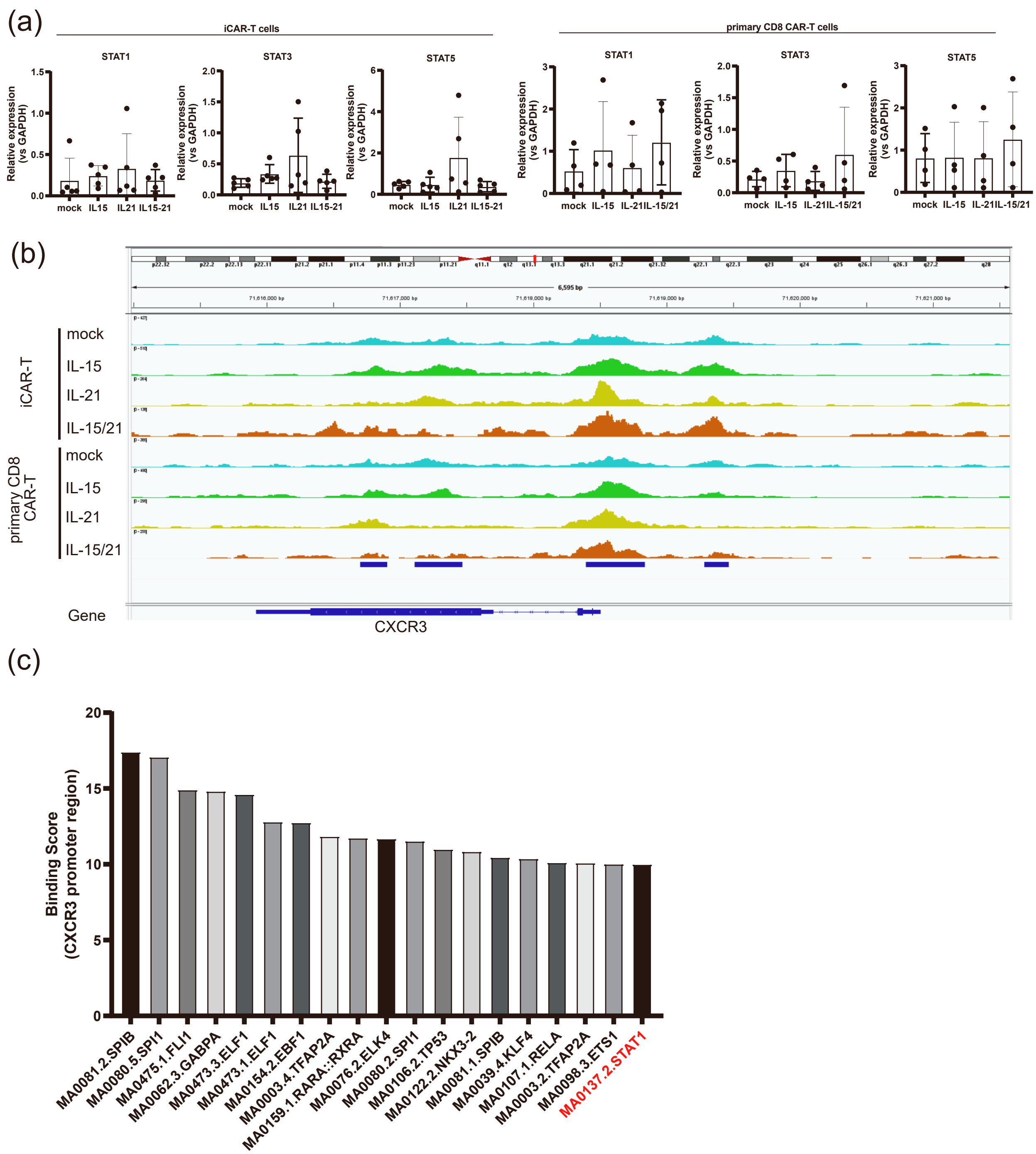


**Figure S6 Chromatin accessibility of CXCR3 region of primary CD8 CAR-T and iCAR-T cells expressing IL-15/21.**

(a) Protein expression of STAT1, 3, and 5 proteins on iCAR-T and primary CD8 CAR-T cells. (b) Chromatin accessibility of each sample near the CXCR3 region. Peaks show chromatin accessibility. Blue bars show the chromatin open region in the genome. Arrows presented in the gene section indicate the direction of the transcription. (c) Transcription factor list of binding CXCR3 upstream chromatin accessibility site by ATAC-seq using JASPAR analysis.

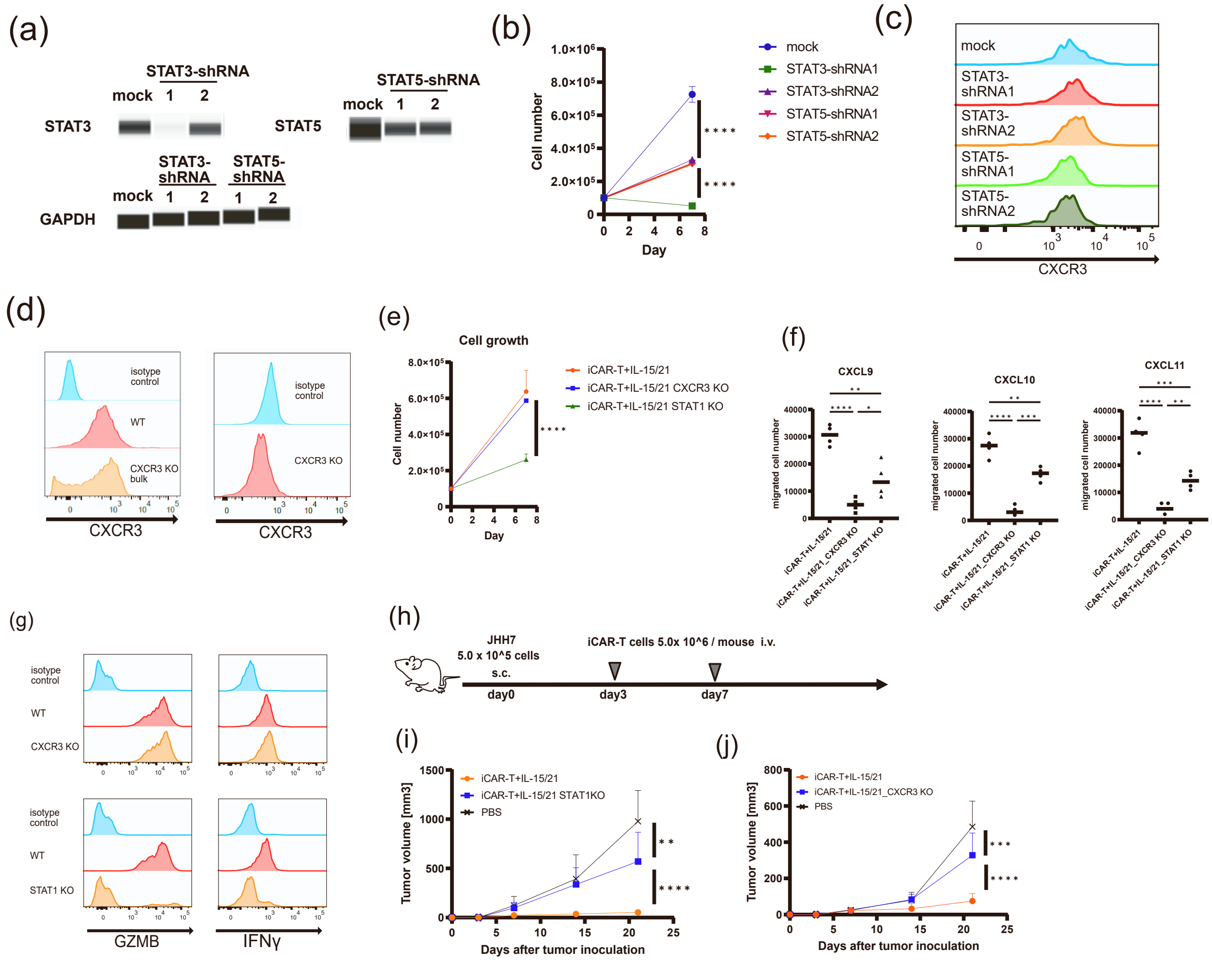


**Figure S7 iCAR-T cells expressing IL-15/21 achieve anti-tumor efficacy through the functional activity of STAT1 and CXCR3.**
(a) Protein expression of STAT 3 and 5 proteins on iCAR-T cells expressing IL-15/21. (b)Representative growth ability on iCAR-T cells expressing IL-15/21 and different shRNA. (c) CXCR3 expression on iCAR-T cells expressing IL-15/21 and different shRNA. (d) CXCR3 expression patterns on CXCR3 KO cells using CRISPR/Cas9 on iCAR-T cells expressing IL-15/21. (e) Representative growth ability on CXCR3 and STAT1 KO iCAR-T cells. (f) Migration activity of iCAR-T cells expressing IL-15/21 against CXCL9, 10, and 11. (g) IFNγ and GZMB expression on CXCR3- or STAT1-KO iCAR-T cells expressing IL-15/21 stimulated with PMA ionomycine. (h) Schedule of the JHH7 cell inoculation and iCAR-T cell injection. 5.0 x $10^5$ JHH7 cells were subcutaneously injected into the left back side of the mice. (i and j) Tumor volume after iCAR-T cell injection.

# Table S1 Pathway analysis using significantly upregulated genes in each sample

| **iCAR-T+mock** |
| --- |
| MECP2 regulates transcription factors |
| ATF4 activates genes in response to endoplasmic reticulum stress |
| PERK regulates gene expression |
| Nuclear signaling by ERBB4 |
| Transcriptional regulation by the AP-2 (TFAP2) family of transcription factors |
| NR1H3 & NR1H2 regulate gene expression linked to cholesterol transport and efflux |
| Signaling by ERBB4 |
| NR1H2 and NR1H3-mediated signaling |
| Defective HEXB causes GM2G2 |
| Transcriptional Regulation by MECP2 |
| Response of EIF2AK4 (GCN2) to amino acid deficiency |
| **iCAR-T+IL-15** |
| Nuclear Receptor transcription pathway |
| Generic Transcription Pathway |
| Gene expression (Transcription) |
| RNA Polymerase II Transcription |
| Diseases of mitotic cell cycle |
| Signaling by ALK fusions and activated point mutants |
| Signaling by ALK in cancer |
| Circadian Clock |
| FOXO-mediated transcription |
| RUNX3 regulates BCL2L11 (BIM) transcription |
| Drug-mediated inhibition of CDK4/CDK6 activity |
| **iCAR-T+IL-21** |
| The role of GTSE1 in G2/M progression after G2 checkpoint |
| Regulation of expression of SLITs and ROBOs |
| Cellular responses to stress |
| Cellular responses to stimuli |
| Viral Infection Pathways |
| Signaling by ROBO receptors |
| Nervous system development |
| Infectious disease |
| Axon guidance |
| Apoptosis |
| Mitotic Anaphase |
| **iCAR-T+IL-15/21** |
| **Immune System** |
| **Cytokine Signaling in Immune system** |
| **Signaling by Interleukins** |
| Interleukin-10 signaling |
| RUNX1 and FOXP3 control the development of regulatory T lymphocytes (Tregs) |
| Transcriptional regulation by RUNX1 |
| MHC class II antigen presentation |
| Transcriptional regulation of white adipocyte differentiation |
| Neutrophil degranulation |
| Interleukin-12 signaling |
| **Chemokine receptors bind chemokines** |
| **primary-CD8T+mock** |
| Attenuation phase |
| HSF1-dependent transactivation |
| Regulation of HSF1-mediated heat shock response |
| Cellular response to heat stress |
| HSF1 activation |
| Cellular responses to stress |
| Cellular responses to stimuli |
| Interferon gamma signaling |
| Cytokine Signaling in Immune system |
| Interferon Signaling |
| Translocation of ZAP-70 to Immunological synapse |
| **primary-CD8T+IL-15/21** |
| Interferon gamma signaling |
| NPAS4 regulates expression of target genes |
| Translocation of ZAP-70 to Immunological synapse |
| Transcriptional Regulation by NPAS4 |
| Phosphorylation of CD3 and TCR zeta chains |
| PD-1 signaling |
| Interferon Signaling |
| Generation of second messenger molecules |
| Cytokine Signaling in Immune system |
| Costimulation by the CD28 family |
| Downstream TCR signaling |

**Table S2 CCL3, CCL3L1, and CCL5 transcription factor analysis (ChIP-Atlas)**

| ChIP-Atlas | | | |
|---|---|---|---|
| **ID** | **Name** | **sample** | **logP-val** |
| SRX831873 | STAT5B | CD8+ T cells | -2.6 |
| SRX831874 | STAT5B | CD8+ T cells | -1.2 |
| SRX831875 | STAT5B | CD8+ T cells | -1.1 |
| SRX831879 | STAT5B | CD8+ T cells | -0.9 |
| SRX831878 | STAT5B | CD8+ T cells | -0.8 |
| SRX9867030 | CTCF | CD8+ T cells | -0.5 |
| SRX9867031 | CTCF | CD8+ T cells | -0.3 |

# Table S3 CCL3, CCL3L1, and CCL5 transcription factor analysis (JASPAR)

| CCL3(JASPAR) | |
|---|---|
| **Name** | **Score** |
| MA0149.1.EWSR1-FLI1 | 33.54873 |
| MA0105.4.NFKB1 | 19.850035 |
| MA0050.2.IRF1 | 19.409983 |
| MA0141.3.ESRRB | 16.922794 |
| MA0137.1.STAT1 | 16.455967 |
| MA0465.2.CDX2 | 16.373117 |
| MA0119.1.NFIC::TLX1 | 16.258879 |
| MA0052.4.MEF2A | 16.13873 |
| MA0496.1.MAFK | 15.688024 |
| MA0481.1.FOXP1 | 15.378537 |
| MA0039.4.KLF4 | 15.1585865 |
| MA0478.1.FOSL2 | 15.048594 |
| MA0148.3.FOXA1 | 14.910424 |
| MA0058.2.MAX | 14.712487 |
| MA0007.2.AR | 14.711017 |
| MA0471.1.E2F6 | 14.676137 |
| MA0036.2.GATA2 | 14.643103 |
| MA0495.1.MAFF | 14.635535 |
| MA0103.2.ZEB1 | 14.586873 |
| CCL3L1(JASPAR) | |
| **Name** | **Score** |
| MA0105.4.NFKB1 | 19.850035 |
| MA0050.2.IRF1 | 19.1672 |
| MA0149.1.EWSR1-FLI1 | 17.427319 |
| MA0141.3.ESRRB | 16.922794 |
| MA0041.2.FOXD3 | 16.74318 |
| MA0481.1.FOXP1 | 16.564342 |
| MA0137.1.STAT1 | 16.455967 |
| MA0465.2.CDX2 | 16.191309 |
| MA0052.4.MEF2A | 16.13873 |
| MA0479.1.FOXH1 | 16.055038 |
| MA0039.4.KLF4 | 15.727278 |
| MA0496.1.MAFK | 15.688024 |
| MA0079.3.SP1 | 15.404602 |
| MA0102.3.CEBPA | 15.34827 |
| MA0025.2.NFIL3 | 15.22379 |
| MA0478.1.FOSL2 | 15.048594 |
| MA0148.3.FOXA1 | 14.910424 |
| MA0499.2.MYOD1 | 14.837332 |
| MA0079.5.SP1 | 14.750445 |
| CCL5(JASPAR) | |
| **Name** | **Score** |
| MA0052.4.MEF2A | 19.489176 |
| MA0060.2.NFYA | 18.788544 |
| MA0162.2.EGR1 | 18.303143 |
| MA0153.2.HNF1B | 17.918585 |
| MA0497.1.MEF2C | 17.788645 |
| MA0502.1.NFYB | 17.639404 |
| MA0007.2.AR | 17.570421 |
| MA0046.2.HNF1A | 17.302526 |
| MA0113.3.NR3C1 | 16.828331 |
| MA0041.2.FOXD3 | 16.75936 |
| MA0050.1.IRF1 | 16.29417 |
| MA0050.2.IRF1 | 16.233736 |
| MA0003.4.TFAP2A | 16.077852 |
| MA0473.1.ELF1 | 16.00517 |
| MA0137.1.STAT1 | 15.944087 |
| MA0495.1.MAFF | 15.8437605 |
| MA0073.1.RREB1 | 15.530028 |
| MA0496.1.MAFK | 15.516559 |
| MA0039.4.KLF4 | 15.439284 |
